# Meningiomas in patients with neurofibromatosis type 2 predominantly comprise ‘immunogenic subtype’ tumours characterised by macrophage infiltration

**DOI:** 10.1186/s40478-023-01645-3

**Published:** 2023-09-26

**Authors:** Yu Teranishi, Satoru Miyawaki, Masahiro Nakatochi, Atsushi Okano, Kenta Ohara, Hiroki Hongo, Daiichiro Ishigami, Yu Sakai, Daisuke Shimada, Shunsaku Takayanagi, Masako Ikemura, Daisuke Komura, Hiroto Katoh, Jun Mitsui, Shinichi Morishita, Tetsuo Ushiku, Shumpei Ishikawa, Hirofumi Nakatomi, Nobuhito Saito

**Affiliations:** 1 Department of Neurosurgery, Faculty of Medicine, The University of Tokyo, 7-3-1 Hongo, Bunkyo-ku, Tokyo 113-8655 Japan; 2 Public Health Informatics Unit, Department of Integrated Health Sciences, Nagoya University Graduate School of Medicine, 1-1-20 Daiko-Minami, Higashi-ku, Nagoya Japan; 3 Department of Neurosurgery, Faculty of Medicine, Kyorin University, 6-20-2 Shinkawa, Mitaka-shi, Tokyo Japan; 4 Department of Pathology, Faculty of Medicine, The University of Tokyo, 7-3-1 Hongo, Bunkyo-ku, Tokyo Japan; 5 Department of Preventive Medicine, Graduate School of Medicine, The University of Tokyo, Tokyo, Japan; 6 Department of Molecular Neurology, Graduate School of Medicine, The University of Tokyo, 7-3-1 Hongo, Bunkyo-ku, Tokyo Japan; 7 Department of Computational Biology and Medical Sciences, Graduate School of Frontier Sciences, The University of Tokyo, Tokyo, Japan

**Keywords:** Neurofibromatosis type 2, Meningioma, Tumour microenvironment, Immune infiltration

## Abstract

Although recent molecular analyses revealed that sporadic meningiomas have various genetic, epigenetic, and transcriptomic profiles, meningioma in patients with neurofibromatosis type 2 (NF2) have not been fully elucidated. This study investigated meningiomas' clinical, histological, and molecular characteristics in NF2 patients. A long-term retrospective follow-up (13.5 ± 5.5 years) study involving total 159 meningiomas in 37 patients with NF2 was performed. Their characteristics were assessed using immunohistochemistry (IHC), bulk-RNA sequencing, and copy number analysis. All variables of meningiomas in patients with NF2 were compared with those in 189 sporadic *NF2*-altered meningiomas in 189 patients. Most meningiomas in NF2 patients were stable, and the mean annual growth rate was 1.0 ± 1.8 cm^3^/year. Twenty-eight meningiomas (17.6%) in 25 patients (43.1%) were resected during the follow-up period. WHO grade I meningiomas in patients with NF2 were more frequent than in sporadic *NF2*-altered meningiomas (92.9% vs. 80.9%). Transcriptomic analysis for patients with NF2/sporadic *NF2*-altered WHO grade I meningiomas (n = 14 vs. 15, respectively) showed that tumours in NF2 patients still had a higher immune response and immune cell infiltration than sporadic *NF2*-altered meningiomas. Furthermore, RNA-seq/IHC-derived immunophenotyping corroborated this enhanced immune response by identifying myeloid cell infiltration, particularly in macrophages. Clinical, histological, and transcriptomic analyses of meningiomas in patients with NF2 demonstrated that meningiomas in NF2 patients showed less aggressive behaviour than sporadic *NF2*-altered meningiomas and elicited a marked immune response by identifying myeloid cell infiltration, particularly of macrophages.

**Supplementary Information:**

The online version contains supplementary material available at 10.1186/s40478-023-01645-3.

## Introduction

Neurofibromatosis type 2 (NF2) is a tumour predisposition syndrome characterised by a benign tumour of the central nervous system [1, 2]. The classical phenotype is presented with bilateral vestibular schwannomas [1, 2]. The phenotype of NF2 is highly diverse because patients may also develop other multiple types of CNS tumours. Meningiomas are the second most frequent type of tumours in NF2 [1]. The frequency of intracranial meningiomas was reported to range from 45 to 58% [2, 3], and the existence of meningiomas in NF2 patients is robustly associated with mortality [4, 5]. Previous studies have delineated the natural history, molecular characteristics, and therapeutic strategies for vestibular schwannomas in NF2 patients [6–9]. However, only a few studies on meningiomas in patients with NF2 have focused on their clinical and histological characteristics [10–14]. According to the latest two reports, meningiomas in NF2 patients are not histologically or clinically more aggressive than sporadic *NF2*-altered meningiomas [11, 12]. However, the question remains as to why meningiomas in NF2 patients show less aggressive behaviour than sporadic *NF2*-altered meningiomas, despite the development of multiple *NF2*-altered meningiomas.

Compared with meningiomas in NF2 patients, recent molecular analyses have established that sporadic meningiomas are not genomically homogenous but have various genetic, epigenetic, and transcriptomic profiles [15–20]. Among the various driver gene mutations in meningiomas, *NF2* alteration is the most commonly-found genetic abnormality in meningiomas, and only sporadic *NF2*-altered meningiomas may present benign, atypical, and malignant tumours [20]. Taking this latest knowledge into consideration, we hypothesize that molecular mechanisms present in NF2 patients to maintain meningiomas at a benign phenotype.

This study investigated the clinical, histological, and molecular characteristics of meningiomas in NF2 patients to decipher the mechanisms of clinical difference between meningiomas in NF2 patients and sporadic *NF2*-altered meningiomas. Herein, we present a long-term follow-up clinical/molecular analysis of meningiomas in NF2 patients compared with that of sporadic *NF2*-altered meningiomas.

## Materials and methods

### Patient population

Data from 85 patients with an established diagnosis of NF2 according to the Manchester NF2 criteria [21, 22] at our institutions, between 2000 and 2019, were used in our analysis (Additional file 1: Figure S1). Twenty-eight patients with incomplete clinical data were excluded. The remaining 58 patients attended our outpatient clinics at least once a year and underwent the diagnostic and treatment procedures when required (Additional file 1: Figure S1). These 58 patients included 53 with de novo NF2, as reported in our previous study [23].

A total of 343 patients with sporadic meningiomas who underwent surgical treatment at The University of Tokyo Hospital between 2000 and 2019 were enrolled in this study (Additional file 1: Figure S1). Patients with incomplete clinical or genetic data or those with NF2 were excluded. The remaining 330 patients were eligible for subsequent analyses (Additional file 1: Figure S1).

### Clinical data

All the clinical data were collected through a retrospective chart review. Clinical endpoints included patient age, sex, and radiological follow-up. Pre- and postoperative radiological data, including tumour size, anatomical location, the extent of resection (EOR), presence/absence and timing of recurrence, were noted. Patients were followed-up with contrast-enhanced MRI (CE-MRI) within 2 days, approximately 6 months, and 1 year after surgery. If there was no tumour recurrence, follow-up with MRI was continued annually. Tumour recurrence was defined as apparent enlargement of the residual tumour on CE-MRI by blind inter-observer agreement between the neuroradiologists and neurosurgeons in charge.

Tumour volumetric analysis was performed using the volumetric function of the OsiriX Lite ver. 9.0 software. The absolute growth rate (cm^3^/year) was calculated using the following formula: (latest tumour size in cm^3^ − initial tumour size in cm^3^)/follow-up interval in years.

### Mutation analysis

The DNA of NF2 patients was obtained from their peripheral blood leucocytes, buccal swabs, hair follicles, and tumour samples. Mutation analysis was performed as previously described methods, including direct Sanger sequencing, multiple ligation-dependent probe amplification (MLPA) (SALSA P044), and targeted deep next-generation sequencing [23]. Data analysis of the targeted deep sequences was performed as described previously [23].

Tumour DNA was extracted from frozen samples using a DNA Extraction Mini Kit (Qiagen, Hilden, Germany) according to the manufacturer's protocol. Mutation and copy number variant (CNV) analyses focusing on 1p/22q loss were performed as previously described, including direct Sanger sequencing, microsatellite analysis, and MLPA (SALSA P044, P088) [17, 23].

### RNA sequencing for WHO grade I meningioma

Total RNA (14 grade I meningiomas in 14 NF2 patients and 15 grade I sporadic *NF2*-altered meningiomas)(Additional file 2: Table S1) was extracted from the same samples using the miRNeasy Mini Kit (Qiagen, Tokyo, Japan) according to the manufacturer's protocols. RNA quality was assessed using an Agilent 4200 Tapestation System (Agilent Technologies, Santa Clara, CA, USA), and libraries for RNA sequencing were prepared using a TruSeq Stranded mRNA Library Prep (Illumina) according to the manufacturer’s protocol. Briefly, the libraries were constructed using 400 ng of total RNA, followed by polyA^+^ RNA isolation, cDNA synthesis, end repair, A-base attachment, and ligation of Illumina's (San Diego, CA, USA) indexed adapters were performed. Library quality was assessed using an Agilent 2100 Bioanalyzer. Library samples were prepared for sequencing using an Illumina NovaSeq 6000 sequencing system with an Illumina NovaSeq 6000 S4 Reagent Kit (Illumina) and sequenced on an Illumina NovaSeq 6000 (150 bp paired-end reads). First, we trimmed the low-quality bases and removed the adapter sequences using the Trimmomatic software. Reads were subsequently mapped to the human reference genome GRCh38 using HISAT2 with default parameters, and the fragments per kilobase of transcript per million mapped reads (FPKM) values for each gene estimate were obtained using StringTie. Human gtf annotation on Ensembl v101 was obtained from Ensembl and used. All transcripts with a variance in FPKM values across the samples of less than one were excluded. All transcripts of *NF2* gene were excluded because of their low variance. Finally, 11,647 genes were remained for further analysis. Principal component analysis was performed based on the gene expression data.

### Differential gene expression analysis in WHO grade I meningioma

Gene expression levels from StringTie between groups were compared using BallGown. The fold-change in the expression level was calculated from the ratio of the mean FPKM of sporadic no/sporadic yes. The significance level of the differential expression analysis was set at a false discovery rate (FDR) q < 0.1. All significant differentially expressed genes were used to construct a heat map. The gene expression profiles of the FPKM values were standardized. Hierarchical clustering was applied into the standardised gene expression profiles based on the Ward D2 linkage method and Euclidean distance using the pheatmap function of the R package.

### Pathway analysis

We searched for pathway enrichment using gene set enrichment analysis (GSEA) and collections of MSigDB v.2023.1 [24]. A rank list was generated by ordering each gene according to the (− 1) × log_10_ (*p* value) × sign(log_2_ (fold change)) from the differential expression analysis. These rank lists were used in the weighted pre-ranked GSEA. Sets of 1000 permutations of the genes were applied to the pre-ranked GSEA performed with the above-described collections of gene sets. An FDR of q < 0.10 was considered significant for the GSEA analysis. The “GSVA” package in R was used to perform GSVA between meningiomas in NF2 patients and sporadic *NF2*-altered meningiomas, using the c7 immunologic signature gene sets as a reference [25]. Hierarchical clustering was applied into the c7 signature profiles based on the Ward D2 linkage method and Euclidean distance using the pheatmap of the R package.

### Estimation of the amount and composition of the immune cell infiltrate

The proportion of immune cell infiltrates subpopulations was estimated using CIBERSORT [26], xCell [27], and ESTIMATE [28] using the mRNA expression data of WHO grade I meningiomas. To perform reference-based deconvolution, we utilised 'LM22 gene signature' for CIBERSORT [26], 'The 489 cell type gene signatures' for xCell [28], and 'A gene list of stromal and immune signature' for ESTIMATE [29]. To estimate tumour purity in ESTIMATE [29], we ran RNA-seq data using the ESTIMATE algorithm, which uses gene expression data to estimate the levels of infiltrating stromal and immune cells and tumour purity.

### Histopathological data

Pathological diagnoses were made by two expert neuropathologists at our institution based on the 2016 WHO classification of tumours of the central nervous system. When the central review was performed, clinical information and index test results were not available for reference by neuropathologists.

Formalin-fixed paraffin-embedded tissue were used for immunohistochemistry (IHC) analysis of 13 meningiomas in NF2 patients and 16 sporadic WHO grade I meningiomas. IHC was performed using whole-slide sections for Ki67, CD3, CD4, CD8, CD19, CD45, CD68, CD163, FOXP3, and Granzyme B. The following antibodies were used: anti-Ki67 rabbit polyclonal (30–9; Ventana Medical Systems, Tucson, AZ), anti-CD3 rabbit monoclonal (1–100; ab10558, Abcam), anti-CD4 rabbit monoclonal (1–100; EPR6855, Abcam), anti-CD8 rabbit monoclonal (1–100; EP1150Y, Abcam), anti-CD19 rabbit monoclonal (1–100; EPR5906, Abcam), anti-CD45 rabbit monoclonal (1–100; ab10558, Abcam), anti-CD68 Mouse monoclonal (1–100; M0814, Dako), anti-CD163 antibody (EPR19518) (ab182422) rabbit, anti-FOXP3 antibody (236A/E7) (ab20034), and anti-granzyme B antibody (GRAN-B-L-CE, clone 11F1). For each antibody, colour deconvolution of the images was performed to obtain separate hematoxylin and DAB images using the colour deconvolution plugin in FIJI (US National Institutes of Health), depending on the pattern of stains (membrane, cytoplasm, or nuclei). The DAB-stained area was obtained in FIJI from setting defined thresholds. IHC was quantified as the average number of positivity per high-power field (HPF) from five distinct regions within each meningioma using the FIJI to account for intra-tumour heterogeneity. The number of cells with further distinction of cell types in percent of total cell count (% TCC).

### Statistical analyses

Statistical analyses were performed using R version 3.6.0 (R Core Team, http://www.R-project.org). Numerical variables are expressed as means and standard deviations. Categorical data were compared between subgroups using Fisher's exact test. The Mann–Whitney U test was used to compare two non-parametric continuous variables. All reported *p* values were two-sided, and in all comparisons, *p* values of less than 0.05 were considered significant.

## Results

### Clinical characteristics

**Fig. 1:**
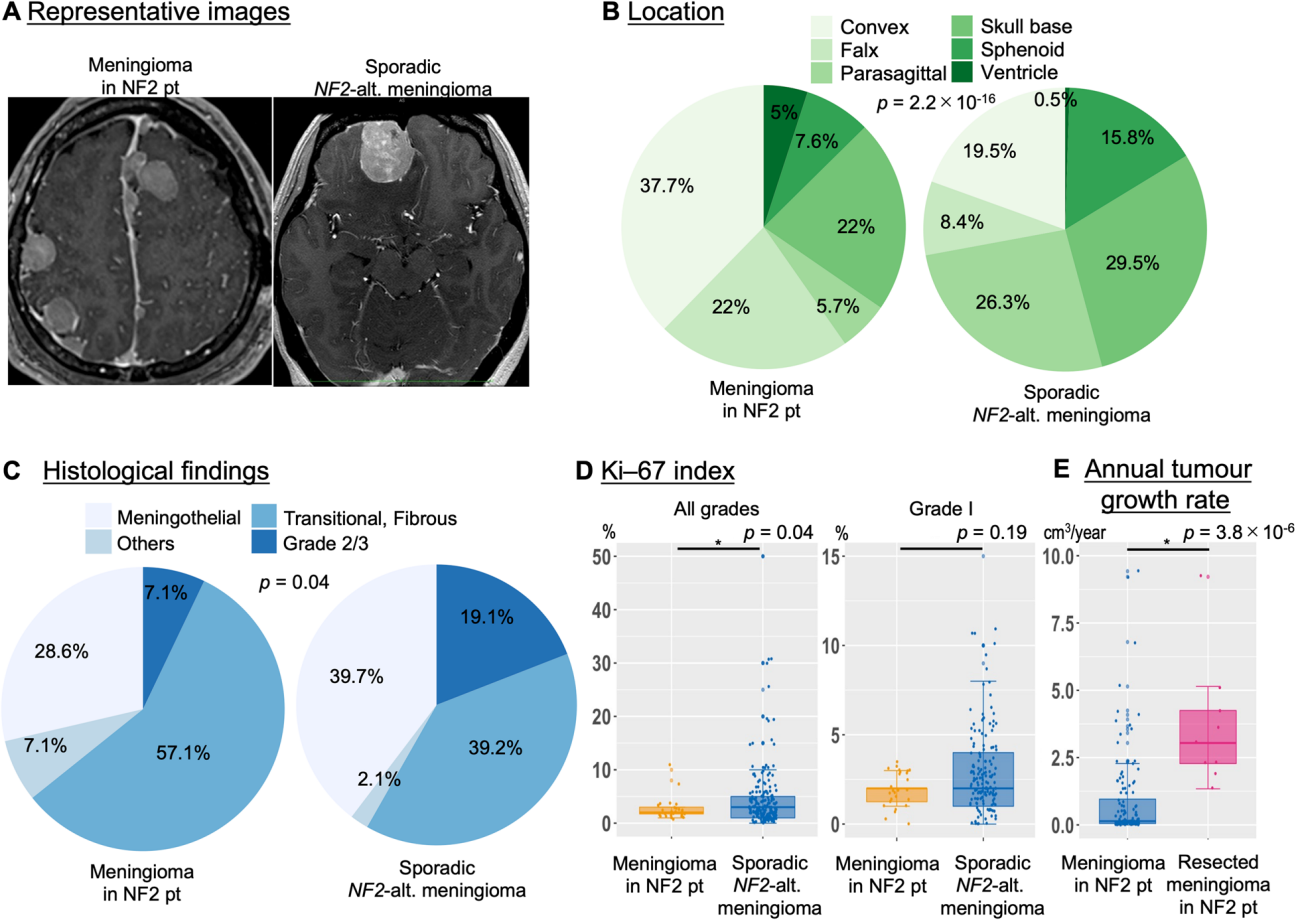
Different clinical characteristics between meningioma in NF2 patients versus sporadic *NF2-*altered meningioma. **A** Representative images of meningiomas in NF2 patients and sporadic *NF2*-altered meningioma. **B** Pie chart of the anatomical location of the meningiomas. **C** Pie chart of the histological findings of the meningiomas. **D** The box plot shows the differences in the Ki-67 index of the meningiomas. **E** The box plot shows the differences in annual tumour growth rate between meningiomas in NF2 patients and operated meningiomas in NF2 patients

A total of 25 de novo meningiomas (15.7%) emerged in 9 patients (24.3%) during the follow-up period, and 37 NF2 patients harboured total 159 meningiomas at the end of the follow-up period (representative case in Fig. 1A, Additional file 1: Figure S1). The average follow-up period in these patients was 13.5 ± 5.5 years (Table 1). Among 37 NF2 patients with meningiomas, germline *NF2* alterations were identified in 18 patients (48.6%), including truncating mutations (9, 24.3%), large deletions (2, 5.4%), splice-site mutations (6, 16.2%), and missense mutations (1, 2.7%) (Table 1). Mosaic NF2 was identified in 11 patients (29.7%) (Table 1). No germline *NF2* alteration or mosaic *NF2* alteration was detected in 8 NF2 patients (21.6%).

**Table 1:** Patient characteristics

Variable	NF2-related (n = 159 tumors, 37 NF2 patients)	Sporadic *NF2*-alterated (n = 189 tumors, 189 patients)	*P* value
Sex	Female: 23 (62.2)	Female: 131 (69.3)	0.44
Follow–up (years)	13.3 ± 5.5	5.5 ± 4.7	6.3 × 10^–12^*
Germline *NF2* alteration	18 (48.6)		
Truncating	9 (24.3)		
Non-truncating	9 (24.3)		
Mosaic NF2	11 (29.7)		
Undetected cases	8 (21.6)		
The number of tumors/pt	4.3	1	1.3 × 10^–35^*
De novo tumors during F/U	25 (17.6) in 9 pt		
Tumor location			2.2 × 10^–16^*
Convexity	60 (37.7)	37 (19.5)	
Falx	35 (22.0)	16 (8.4)	
Parasagittal	9 (5.7)	50 (26.3)	
Skull-base, tentorial	35 (22.0)	56 (29.5)	
Sphenoidal	12 (7.6)	30 (15.8)	
Ventricle	8 (5.0)	1 (0.5)	
Tumor volume	13.4 ± 30.3	14.8 ± 12.7	4.0 × 10^–6^*
Tumor growth rate	1.0 ± 1.8		
*Operated tumor*	n = 28	n = 189	
Age at the surgery	42.5 ± 16.8	60.3 ± 13.2	4.1 × 10^–8^*
WHO histological grade			0.04*
Meningothelial	8 (28.6)	75 (39.7)	
Transitional/fibrous	16 (57.1)	74 (39.2)	
Others	2 (7.1)	4 (2.1)	
Grade II/III	2 (7.1)	36 (19.1)	
Ki-67 index	2.4 ± 1.9	5.1 ± 7.6	0.04*

These tumours were located in the convexity (37.7%), the falx (22.0%), the parasagittal (5.7%), the skull-base/tentorial (22.0%), the sphenoidal (7.6%), and the ventricles (5.0%) (Fig. 1B, Table 1). There was no difference of meningiomas between the initial tumours and the later developing tumors.

Among 330 sporadic meningiomas identified during the follow-up periods, molecular analysis revealed 189 sporadic *NF2*-altered meningiomas with *NF2* mutations and/or 22q loss (representative case in Fig. 1A). These tumours were located in the convexity (19.5%), the falx (8.4%), the parasagittal (26.3%), the skull-base/tentorial (29.5%), the sphenoidal (15.8%), and the ventricles (0.5%) (Fig. 1B, Table 1).

The anatomical distribution of meningiomas differed between NF2 patients and sporadic case with *NF2* alterations, especially in the frequency of the falx meningiomas (22.0% in meningiomas of NF2 patients, 8.4% in sporadic *NF2*-altered tumours, *p* = 2.2 × 10^–16^) (Table 1).

### Surgical outcome and histological findings

Of the 159 meningiomas in NF2 patients, 28 meningiomas (17.6%) in 25 patients (43.1%) were resected during the follow-up period. The age at the surgery in NF2 patients was younger than that in sporadic *NF2*-altered meningiomas (42.5 ± 16.8 vs. 60.3 ± 13.2, *p* = 4.1 × 10^–8^) (Table 1). 92.9% were WHO grade I (meningothelial, 28.6%; transitional/fibrous, 57.1%; others, 7.1%) and 7.1% were WHO grade II/III (Fig. 1C, Table 1). Of the 189 sporadic *NF2*-altered meningiomas, 80.9% were WHO grade I (meningothelial: 39.7%, transitional/fibrous: 39.2%, others: 2.1%) and 19.1% were WHO grade II/III (Fig. 1C, Table 1). The frequency of WHO grade I meningiomas differed between tumour in NF2 patients and sporadic *NF2*-altered tumours (*p* = 0.04). Univariate and multivariate analyses for Grade II/III *NF2*-altered meningiomas showed that high Ki–67 index and male sex were predictors of high-grade meningiomas, and the germline *NF2* alteration did not represent a significant predictive factor (Additional file 2: Table S2).

### Tumour behaviour

The mean tumour volume at diagnosis was 13.4 ± 30.3 cm^3^ in all meningiomas with NF2 patients, and 33.2 ± 34.0 cm^3^ at diagnosis in resected meningiomas with NF2 patients (Table 1). The mean annual growth rate (cm^3^/year) was 1.0 ± 1.8 in all meningiomas with NF2 patients and 3.7 ± 2.4 in resected meningiomas with NF2 patients (Fig. 1E, *p* = 3.8 × 10^–6^).

### Copy number analysis for meningiomas in NF2 patients and sporadic *NF2*-altered meningiomas

**Fig. 2:**
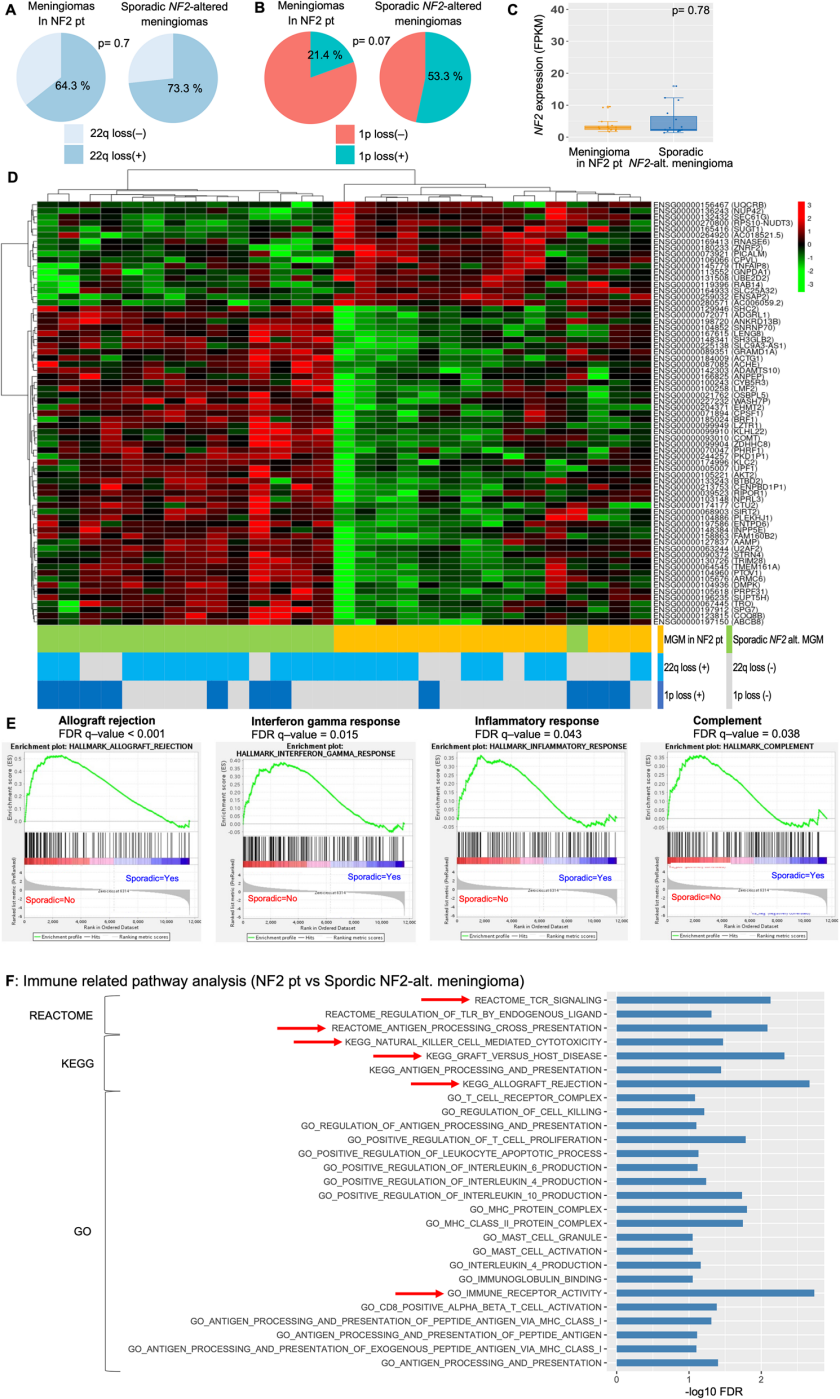
Molecular characteristics of *NF2*-altered WHO grade I meningiomas (Meningioma in NF2 patients versus sporadic *NF2*-altered meningiomas). **A** Pie chart of the frequency of chromosome 22 q loss in the meningiomas. **B** Pie chart of the frequency of chromosome 1 p loss in the meningiomas. **C** The box plot shows the differences in the meningiomas’ *NF2* expression (FPKM). **D** Unsupervised hierarchical clustering of genes revealed two molecular groups that mostly matched meningiomas in patients with NF2 and sporadic *NF2*-altered meningiomas **E** GSEA showed that the differentially expressed genes between NF2 patients’ and sporadic *NF2*-altered tumours were significantly overrepresented in signatures associated with ‘ALLOGRAFT REJECTION’, ‘INTERFERON GAMMA RESPONSE’, ‘COMPLEMENT’, and ‘INFLAMMATORY RESPONSE’. **F** Pathway analysis of differentially expressed genes revealed that the immune response-associated genes were the most significantly enriched transcripts in NF2 patients’ relative to sporadic *NF2*-altered tumours

CNV analysis showed that 22q loss was found in 69% (64.3% of NF2 patients’, 73.3% of sporadic *NF2*-altered meningiomas, *p* = 0.7) (Fig. 2A). With respect to 1p loss, 21.4% of NF2 patients’ and 53.3% of sporadic *NF2*-altered tumour showed 1p loss (*p* = 0.07) (Fig. 2B).

### Immune cell expression in meningiomas with NF2 patients shown in RNA sequence transcriptomes

To characterise the specific transcriptional profile that affect the different clinical phenotypes of meningiomas in NF2 patients compared to sporadic *NF2*-altered meningiomas, we first investigated the messenger RNA (mRNA) expression profiles of 29 primary WHO grade I meningiomas (14 grade I meningiomas in 14 NF2 patients versus 15 grade I sporadic *NF2*-altered meningiomas) (Additional file 1: Figures S1, S2A-C, Additional file 2: Table S3). The RNA sequencing confirmed equally low *NF2* expression in patients with NF2 and sporadic *NF2*-altered meningiomas (Fig. 2C, *p* = 0.78). Unsupervised hierarchical clustering of genes revealed two molecular groups that mostly matched meningiomas in patients with NF2 and sporadic *NF2*-altered meningiomas (Fig. 2D).

We subsequently performed GSEA using the hallmark collection in MSigDB v.2023.1 and found that the differentially expressed genes in NF2 patients’ compared to sporadic *NF2*-altered tumours were significantly overrepresented in signatures associated with 'Allograft rejection' (FDR q-value < 0.001), 'Interferon gamma response' (FDR q-value = 0.015), 'Inflammatory response' (FDR q-value = 0.043), and 'Complement' (FDR q-value = 0.038) (Fig. 2E, Additional file 2: Table S4). Thereafter, the 29 *NF2*-altered WHO grade I meningiomas were individually compared by performing unsupervised hierarchical clustering of the tumours based on significant immunological signature gene sets with GSVA analysis, revealing two molecular clusters that mostly matched meningiomas in patients with NF2 and sporadic *NF2*-altered meningiomas (Additional file 1: Figure S2D, Additional file 2: Table S5).

Gene Ontology terms closely related to ‘TCR SIGNALLING’ (− log_10_ FDR = 2.13), ‘ANTIGEN PROCESSING CROSS PRESENTATION’ (− log_10_ FDR = 2.09), ‘GRAFT VERSUS HOST DISEASE’ (− log_10_ FDR = 2.32), ‘ALLOGRAFT REJECTION’ (− log_10_ FDR = 2.67), ‘IMMUNE RECEPTOR ACTIVITY’ (− log_10_ FDR = 2.73), and ‘NATURAL KILLER CELL MEDIATED CYTOTOXICITY’ (− log_10_ FDR = 1.47) were significantly enriched in NF2 patients (Fig. 2F). The mRNA expression of immune-related genes was compared between NF2 and non-NF2 patients, patients with 1p loss and non-carriers, and patients with recurrence (+) (Additional file 1: Figure S4).

We compared the mRNA expression profiles of meningiomas between germline NF2 patients (5 tumours) and mosaic NF2 patients (9 tumours). The resulting MA plot showed no difference in mRNA expression profiles between germline NF2 patients and mosaic NF2 patients (Additional file 1: Figure S3).

### Immune cell expression in meningiomas with NF2 patients estimated by deconvoluted data

**Fig. 3:**
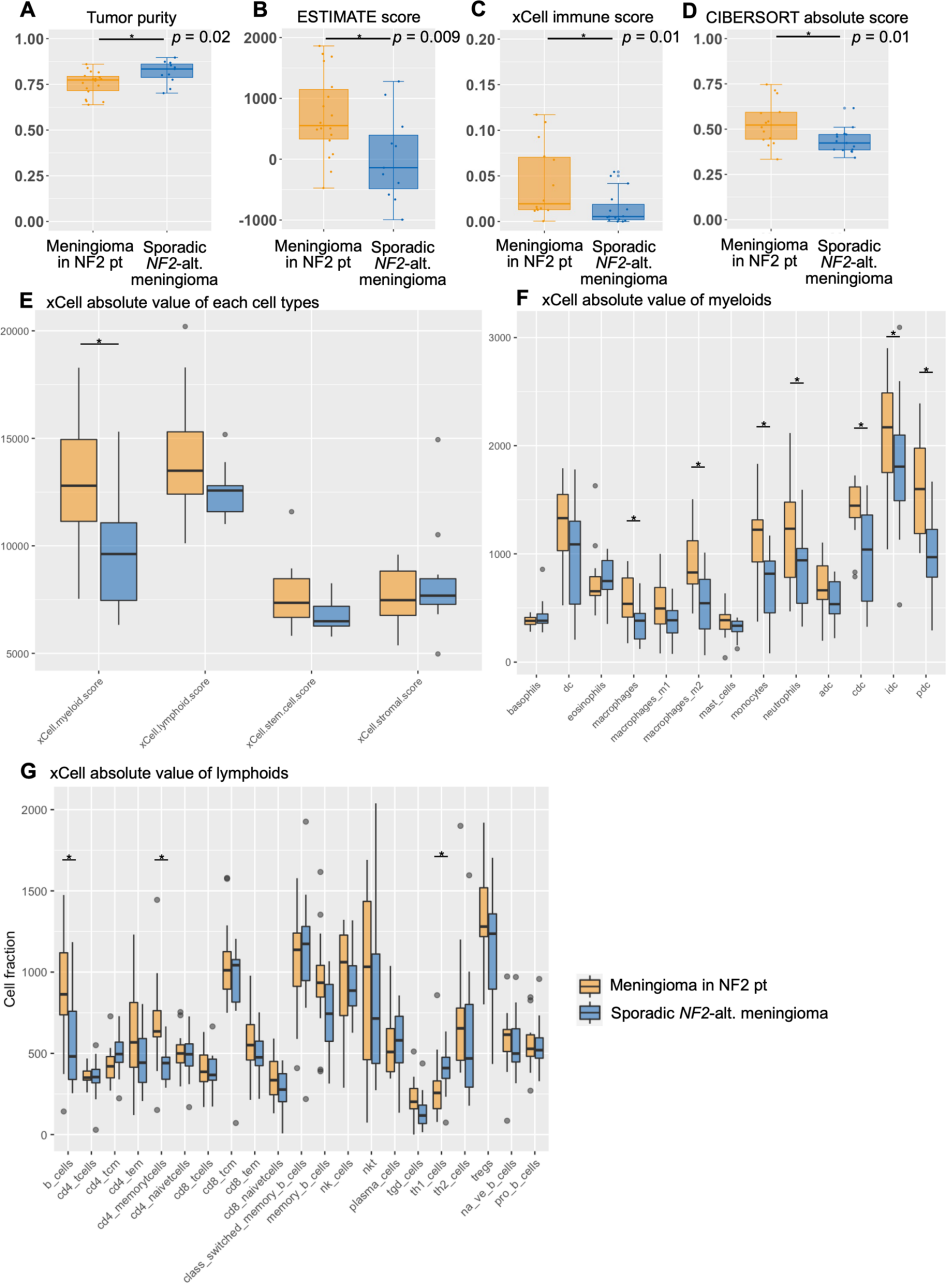
Immune infiltration and immune activity in *NF2*-altered meningiomas The RNA-seq–derived immune metrics from three methods including xCell^27^, ESTIMATE^28^, and CIBERSORT^26^ was compared between meningiomas in NF2 patients and sporadic *NF2*-altered meningioma **A** ESTIMATE tumour purity, **B** ESTIMATE immune score, **C** xCell immune score, **D** CIBERSORT immune score, **E** xCell absolute value of each cell types, **F** xCell absolute value of myeloid. dc: Dendritic cells, adc: activated dendritic cells, cdc: Conventional dendritic cells, idc: Immature dendritic cells, pdc: Plasmcytoid dendrtic cells. **G** xCell absolute value of lymphoid

To clarify immune infiltration and activity in NF2 patients’ tumours, we next quantified and deconvolved the immune infiltration using several methods, including xCell [27], ESTIMATE [28], and CIBERSORT [26]. The inference of the fraction of immune cells and consequently the tumour cell purity within tumour samples showed that the meningiomas in NF2 patients had significantly lower tumour purity (74.4% ± 7.3 in NF2 patients, 80.9% ± 5.2 in sporadic *NF2*-altered meningiomas, *p* = 0.02) and higher immune score (ESTIMATE score;* p* = 0.009, xCell immune score;* p* = 0.01, CIBERSORT absolute score;* p* = 0.01) when compared with sporadic *NF2*-altered meningiomas (Fig. 3A-D, Additional file 1: S5). Among the expressed immune cell types, the xCell [27] results showed a higher absolute value of myeloid cells in NF2 patients than in sporadic *NF2*-altered tumours (*p* = 0.008) (Fig. 3E-G, Additional file 1: Figure S6). In terms of detailed cell type of expression based on the deconvoluted data, myeloid (macrophage;* p* = 0.02, M2;* p* = 0.005, monocytes;* p* = 0.002, neutrophils;* p* = 0.03, conventional dendritic cell (CDC);* p* = 0.007, plasmacytoid dendritic cell (PDC);* p* = 0.001) and lymphoid cells (B-cells;* p* = 0.02, CD4^+^ memory T-cells;* p* = 0.002, gamma delta T cell (tgd cells); *p* = 0.04) expressed higher in the tumour in NF2 patients than sporadic *NF2*-altered meningiomas (Fig. 3E–G). Deconvoluted data were also compared between patients carrying 1p loss, non-carriers, and patients with recurrence (+) (Additional file 1: Figures S5, 6). Consistent with these data, GSEA analysis using single-cell data (C8: cell type signature gene set) showed that the differentially expressed genes in NF2 patients compared to sporadic *NF2*-altered tumours were significantly overrepresented in signatures associated with ‘DESCARTES FETAL INTESTINE MYELOID CELLS’ (FDR q-value < 0.001), ‘DESCARTES FETAL CEREBELLUM MICROGLIA’ (FDR q-value < 0.001), and ‘CUI DEVELOPING HEART C8 MACROPHAGE’ (FDR q-value < 0.001) (Additional file 1: Figure S7, Additional file 2: Table S6). Furthermore, GSEA analysis using single-cell data (C8: cell type signature gene set) showed the differentially expressed genes in non-recurrent tumours compared to recurrent tumours were significantly overrepresented in signatures associated with ‘TRAVAGLINI LUNG PROLIFERATING NK T-CELL’ (FDR q-value = 0.026), and ‘HE LIM SUN FETAL LUNG C4 ACTIVATED NK-CELL’ (FDR q-value = 0.034) (Additional file 1: Figure S8, Additional file 2: Table S7).

### Immune cell infiltration in meningiomas with NF2 determined by IHC

**Fig. 4:**
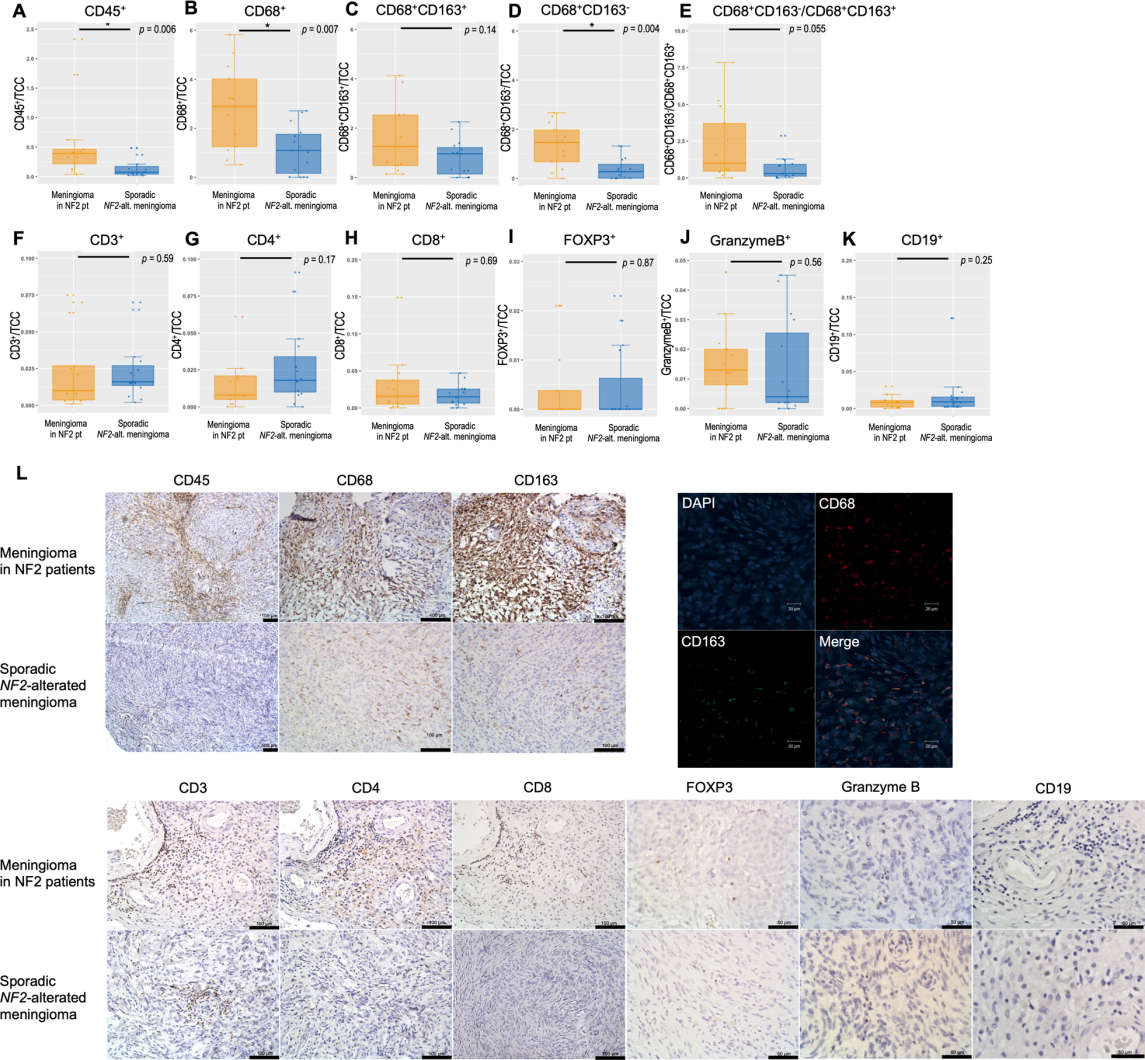
Quantification of immune cell infiltration between NF2 patients’ and sporadic *NF2*-altered tumours. Immunohistochemistry was performed using whole slide sections for CD3, CD4, CD8, CD19, CD45, CD68, CD163, FOXP3, and Granzyme B. IHC was quantified as the average number of nuclei/cell positivity per high-power field (HPF) from five distinct regions within each meningioma using the colour deconvolution plugin in FIJI (**A**; CD45, **B**; CD68^+^, **C**; CD68^+^163^−^, **D**; CD68^+^163^+^, **E**; CD68^+^163^−^ macrophages/CD68^+^163^+^ macrophages, **F**; CD3, **G**; CD4, **H**; CD8, **I**; FOXP3, **J**; Granzyme B, **K**; CD19^+^). **L** Representative IHC/IF images with each antibody

We subsequently evaluated the presence of immune cell infiltrates in the tumour microenvironments of *NF2*-altered meningiomas by quantitative immunostaining for the immune cell markers CD3, CD4, CD8, CD19, CD45, CD68, CD163, FOXP3, and granzyme B (Fig. 4A–L, Additional file 1: Figures S9, 13). IHC staining supported the findings from mRNA sequencing results, identifying more abundant CD45^+^ leukocytes (3.0% ± 1.7/TCC, *p* = 0.006) (Fig. 4A), CD68^+^ macrophages (2.8% ± 1.7 /TCC, *p* = 0.007) (Fig. 4B), and CD68^+^163^−^ macrophages in meningiomas with NF2 patients (1.3% ± 0.9/TCC, *p* = 0.004) (Fig. 4D) infiltrated in NF2 patients than sporadic *NF2*-altered meningiomas. Other immune cells labelled with CD3, CD4, CD8, CD19, FOXP3, and granzyme B did not show different infiltrations between meningiomas in NF2 patients and sporadic *NF2*-altered cases (Fig. 4). We observed very few cells showing FOXP3, granzyme B, and CD19 signals (Fig. 4I, J, K), which is consistent with previous reports [29].

### Comparison of RNA-seq–derived and IHC-derived immunophenotyping

To determine whether these RNA-seq-derived metrics of immune infiltration were consistent with one another and with the histological assessment of immune cell infiltration in NF2 patients and sporadic *NF2*-altered tumours, we systematically examined the intermethod agreement of each of these techniques.

In the meningiomas analysed in NF2 patients, the RNA-seq-derived immune metrics (CIBERSORT [26], xCell [27], and ESTIMATE [28]) were strongly correlated with one another (Spearman r = 0.56–0.85, *p* < 0.001, Additional file 1: Figure S11).

We found that IHC-derived and RNA-seq–derived measures of leukocyte infiltration (CD45 cells vs. CIBERSORT absolute score [26], and ESTIMATE immune score [28]) were significantly correlated (Spearman r = 0.46 – 0.5, *p* = 5.9 × 10^–4^ – 0.01, Additional file 1: Figure S11), demonstrating a high validity of RNA-seq.

## Discussion

Whether meningiomas in NF2 patients are histologically more malignant than sporadic meningiomas [10–14] remains controversial. While previous reports have performed clinical and histological analyses of meningiomas in NF2 patients compared with those of sporadic meningiomas [10–14], our study uniquely focused on the comparison of NF2 patients’ meningiomas with sporadic *NF2*-altered meningiomas. Our clinical evaluation demonstrated that most meningiomas in NF2 patients (n = 159) were stable and that the mean annual growth rate (cm^3^/year) was 1.0 ± 1.8. This mean annual growth rate was less than 2 cm^3^/year, described as the slow-growth group in the AIMSS score by Lee et al. [30]. Regarding WHO histological grade, the frequency of WHO grade I meningiomas in NF2 patients’ tumours was 92.1% (80.9% in sporadic *NF2*-altered meningiomas; *p* = 0.04). Accordingly, contrary to previous reports [13, 14], our results clearly showed that meningiomas in NF2 patients are not histologically or clinically more aggressive than sporadic *NF2*-altered meningiomas, which is in line with the latest two reports [11, 12]. However, the question remains as to why meningiomas in NF2 patients show less aggressive behaviour than sporadic meningiomas, despite the development of multiple *NF2*-altered meningiomas. According to the latest two reports, one reasonable explanation for this is that *NF2* predisposes patients to the development of meningiomas that then have independent growth rates or clinical outcomes [11, 12]. Although a recent study on sporadic *NF2*-altered meningioma showed that transcriptomic changes, multiple CNVs, hypermethylated status, and immune cell infiltration clearly affect clinical tumour behaviour [16, 18, 20], meningiomas in patients with NF2 have been scarcely evaluated.

We addressed this issue by molecular background analysis of meningiomas in NF2 patients with bulk RNA-seq. To the best of our knowledge, no study has evaluated the tumour-microenvironment of meningiomas in NF2 patients using transcriptomic analysis. Our results showed that meningiomas in NF2 patients have high immune activity, as identified by identifying myeloid cell infiltration, especially by macrophages. The latest integrated molecular classification of sporadic meningiomas classifies them into four molecular groups (immunogenic, benign *NF2* wild-type, hypermetabolic, and proliferative) [20]. The immunogenic group consisted mainly of *NF2*-altered meningiomas and showed benign clinical outcomes. In contrast, the hypermetabolic and proliferative groups are also mainly *NF2*-altered and clinically malignant tumours [20]. Consistent with this classification, other studies demonstrated that sporadic benign *NF2*-altered meningiomas showed an immune-rich status characterised by macrophages infiltration, and that some *NF2*-altered meningiomas have an immune-poor status characterized by clinically malignant outcomes despite being WHO grade I meningioma [31–35].

Our study showed that most grade I *NF2*-altered meningiomas showed immune cell infiltration, which is consistent with the latest reports. When comparing immune cell infiltration between NF2 patients and sporadic *NF2*-altered meningiomas, the tumours of NF2 patients showed higher immune infiltration than those observed in sporadic tumours. However, these results require careful interpretation. The bar graph for IHC in each case shows that many cases also showed rich immune cell infiltration in sporadic *NF2*-altered meningiomas (Additional file 1: Figure S10). Only 4 cases presented with no macrophage infiltration in sporadic *NF2*-altered meningiomas. Hence, our findings suggest that sporadic *NF2*-altered grade I meningioma mainly comprises immune-rich tumours, although some tumours are immune-poor. In contrast, meningiomas in NF2 patients predominantly comprise the immunogenic group with macrophage infiltration. We also compared the mRNA expression of related proteins with that of immunogenic meningioma [20] and found that tumours in NF2 patients also showed higher expression of these mRNA than sporadic *NF2*-altered tumours (Additional file 1: Figure S12).

The significance of the high immune activity of macrophages in meningioma tumour behaviour remains unclear. High immune activity in sporadic *NF2*-altered meningiomas has only been reported by the latest studies using single nuclear/cell RNA sequencing [20, 32–35]. These reports state that high immune activity, characterised by rich macrophage infiltration, is observed only in benign meningiomas and not in progressive meningiomas. However, no study has clarified what this high immune activity with rich macrophages indicates in tumour behaviour. An integrated pathway analysis based on single nuclear RNA sequencing data reported by Blume et al. [32] showed that macrophages in benign *NF2*-altered meningiomas could activate NK cells to prevent rapid tumour growth, as observed in high-grade meningiomas. Consistent with this, high activity of NK cells was observed in meningiomas of NF2 patients and in non-recurrent cases in our study (Figs. 2F, 3G, S6, S7, S8, S9). By corroborating these findings with the latest data and our results, we speculate that higher immune activity, including rich macrophages and NK T-cell activity, may contribute to the less aggressive tumour behaviour of meningiomas in NF2 patients.

Our study had several limitations that should be addressed in future studies. First, it had a small sample size and a retrospective, single-institution design, which restricted the variables for the assessment of those included in the database. Charts were reviewed retrospectively; thus, not all the clinical and genomic data could be collected. Although *TERT* promoter mutations and *CDKN2A/B* deletions are known to confer WHO grade III meningiomas in the latest WHO grades for CNS tumours, we did not evaluate the respective molecular analyses (*TERT* promoter and *CDKN2A/B* deletion) and utilised the 2016 WHO classifications. Regarding histological findings, the frequency of transitional/fibrous meningioma in our cases (Fig. 1C: 57.1% in NF2 patients and 39.2% in sporadic *NF2*-altered meningiomas) was found to be relatively lower than that in previous papers [12]; however, further studies on a multi-centre, larger cohort are needed to avoid bias.

Our study was designed to reveal the clinical and molecular characteristics of meningiomas in NF2 patients, but not the immune mechanisms underlying meningioma behaviour. Furthermore, we did not compare NF2-patients' meningiomas with other subtypes of sporadic meningiomas. To clarify this, further studies using comprehensive molecular analyses, including DNA methylation analysis and single-cell RNA sequence, of all subtypes of meningiomas are required.

In conclusion, by conducting clinical, histological, and transcriptomic analyses of meningiomas in NF2 patients, we demonstrated that meningiomas in NF2 patients showed less aggressive behaviour than sporadic *NF2*-altered meningiomas and elicited marked immune responses by identifying myeloid cell infiltration, particularly in macrophages.

### Supplementary Information

**Additional file 1.** **Figure S1**. Flow Chart in this study; **Figure S2**.A: The MA plot based on the RNA sequencing in NF2 patients and sporadic NF2-altered meningiomas. B: The volcano plot based on the RNA sequencing in NF2 patients and sporadic NF2-altered meningiomas. C: The principal component analysis based on the RNA sequencing in NF2 patients and sporadic NF2-altered meningiomas. D: Gene set variation analysis (GSVA) based on c7 immunologic signature gene sets clearly distinguished 2 clusters; **Figure S3**. The MA plot based on the RNA sequencing in germline NF2 patients and mosaic NF2 patients; **Figure S4**. A: Each immunologic gene expression in NF2 patients and sporadic NF2-altered meningiomas.B: Each immunologic gene expression in ‘1p loss (-)’ and ‘1p loss (+)’. C: Each immunologic gene expression in ‘recurrence (-)’ and ‘recurrence (+)’; **Figure S5**. Deconvoluted score using CIBERSORT, xCell, and ESTIMATE. A: Each deconvoluted score in NF2 patients and sporadic NF2-altered meningiomas. B: Each deconvoluted score in ‘1p loss (-)’ and ‘1p loss (+)’. C: Each deconvoluted score in ‘recurrence (-)’ and ‘recurrence (+)’; **Figure S6**. Infiltrated cells based on deconvoluted data. A: Each infiltrated cell in NF2 patients and sporadic NF2-altered meningiomas. B: Each infiltrated cell in ‘1p loss (-)’ and ‘1p loss (+)’. C: Each infiltrated cell in ‘recurrence (-)’ and ‘recurrence (+)’; **Figure S7**. GSEA using single-cell data (C8): NF2 vs sporadic; **Figure S8**. GSEA using single-cell data (C8): non-recurrence vs recurrence; **Figure S9**. Quantification of immune cell infiltration by IHC. A: Quantification of immune cells in “1p loss (-)” and ”1p loss (+)”. B: Quantification of immune cells in ‘recurrence (-)’ and ‘recurrence (+)’; **Figure S10**. Quantification of immune cell infiltration by IHC in each case; **Figure S11**. A-C: The correlation analysis of each RNA-seq–derived immune metrics (CIBERSORT vs ESTIMLATE [A], xCell vs CIBERSORT [B], and ESTIMATE vs xCell [C]). D,E: The correlation analysis of IHC-derived and RNA-seq–derived measures of leukocyte infiltration (CD45 cells vs CIBERSORT absolute score [D], and ESTIMATE immune score [E]); **Figure S12**. Gene expression regarding ‘immunogenic subtype’ of meningiomas.

**Additional file 2.** **Table S1**. Patient Characteristics of samples for RNA seq; **Table S2**. Univariate/Multivariate Odds ratio for Grade II/III in NF2-mutated meningiomas; **Table S3**.Significantly differentially expressed genes; **Table S4**. Significantly enriched hallmark gene sets identified in the GSEA; **Table S5**. Significantly enriched immunologic signature gene sets identified in the GSVA; **Table S6**. Significantly enriched C8 gene sets identified in the GSEA between NF2 vs Sporadic; **Table S7**. Significantly enriched C8 gene sets identified in the GSEA betweem no recurrence vs recurrence [E]).

## Data Availability

The normalized count data of RNAseq are available at the Gene Expression Omnibus under the following accession number: GSE232528.

## Abbreviations

CNV: Copy number variant
EOR: Extent of resection
FDR: False discovery rate
GSEA: Gene set enrichment analysis
GSVA: Gene set variation analysis
HPF: High-power field

## References

[1] Evans DGR (2009). Neurofibromatosis type 2 (NF2): a clinical and molecular review. Orphanet J Rare Dis.

[2] Asthagiri AR, Parry DM, Butman JA, Kim HJ, Tsilou ET, Zhuang Z (2009). Neurofibromatosis type 2. Lancet.

[3] Smith MJ, Higgs JE, Bowers NL, Halliday D, Paterson J, Gillespie J (2011). Cranial meningiomas in 411 neurofibromatosis type 2 (NF2) patients with proven gene mutation: clear positional effect of mutations, absence of female severity effect on age at onset. J Med Genet.

[4] Baser ME, Friedman JM, Aeschliman D, Joe H, Wallace AJ, Ramsden RT (2002). Predictors of the risk of mortality in neurofibromatosis 2. Am J Hum Genet.

[5] Hexter A, Jones A, Joe H, Heap L, Smith MJ, Wallace AJ (2015). Clinical and molecular predictors of mortality in neurofibromatosis 2: a UK national analysis of 1192 patients. J Med Genet.

[6] Lewis D, Donofrio CA, O'Leary C, Li KL, Zhu X, Williams R (2020). The microenvironment in sporadic and neurofibromatosis type II–related vestibular schwannoma: the same tumor or different? A comparative imaging and neuropathology study. J Neurosurg.

[7] Samii M, Matthies C, Tatagiba M (1997). Management of vestibular schwannomas (acoustic neuromas): auditory and facial nerve function after resection of 120 vestibular schwannomas in patients with neurofibromatosis 2. Neurosurgery.

[8] Brackmann DE, Fayad JN, Slattery WH, Friedman RA, Day JD, Hitselberger WE (2001). Early proactive management of vestibular schwannomas in neurofibromatosis type 2. Neurosurgery.

[9] Peyre M, Goutagny S, Imbeaud S, Bozorg-Grayeli A, Felce M, Sterkers O (2011). Increased growth rate of vestibular schwannoma after resection of contralateral tumor in neurofibromatosis type 2. Neuro Oncol.

[10] Goutagny S, Kalamarides M (2010). Meningiomas and neurofibromatosis. J Neurooncol.

[11] Abi Jaoude S, Peyre M, Degos V, Goutagny S, Parfait B, Kalamarides M (2020). Validation of a scoring system to evaluate the risk of rapid growth of intracranial meningiomas in neurofibromatosis type 2 patients. J Neurosurg.

[12] Goutagny S, Bah AB, Henin D, Parfait B, Grayeli AB, Sterkers O (2012). Long-term follow-up of 287 meningiomas in neurofibromatosis type 2 patients: clinical, radiological, and molecular features. Neuro Oncol.

[13] Perry A, Giannini C, Raghavan R, Scheithauer BW, Banerjeee R, Margraf L (2001). Aggressive phenotypic and genotypic features in pediatric and NF2- associated meningiomas: a clinicopathologic study of 53 cases. J Neuropathol Exp Neurol.

[14] Antinheimo J, Haapasalo H, Halite M, Tatagiba M, Thomas S, Brandis A (1997). Proliferation potential and histological features in neurofibromatosis 2-associated and sporadic meningiomas. J Neurosurg.

[15] Clark VE, Erson-Omay EZ, Serin A, Yin A, Cotney J, Ozduman K (2013). Genomic analysis of non-NF2 meningiomas reveals mutations in TRAF7, KLF4, AKT1, and SMO. Science.

[16] Sahm F, Schrimpf D, Stichel D, Jones DTW, Heilscher T, Schefzyk S (2017). DNA methylation—based classification and grading system for meningioma: a multicentre, retrospective analysis. Lancet Oncol.

[17] Okano A, Miyawaki S, Hongo H, Dofuku S, Teranishi Y, Takayanagi S (2021). Associations of pathological diagnosis and genetic abnormalities in meningiomas with the embryological origins of the meninges. Sci Rep.

[18] Prager BC, Vasudevan HN, Dixit D, Bernatchez J, Wu Q, Wallace LC (2020). The meningioma enhancer landscape delineates novel subgroups and drives druggable dependencies. Cancer Discov.

[19] Boetto J, Bielle F, Sanson M, Peyre M, Kalamarides M (2017). SMO mutation status defines a distinct and frequent molecular subgroup in olfactory groove meningiomas. Neuro Oncol.

[20] Nassiri F, Liu J, Patil V, Mamatjan Y, Wang JZ, Hugh-White R (2021). A clinically applicable integrative molecular classification of meningiomas. Nature.

[21] Evans DG, Huson SM, Donnai D, Neary W, Blair V, Teare D (1992). A genetic study of type 2 neurofibromatosis in the United Kingdom. I. Prevalence, mutation rate, fitness, and confirmation of maternal transmission effect on severity. J Med Genet.

[22] Smith MJ, Bowers NL, Bulman M, Gokhale C, Wallace AJ, King AT (2017). Revisiting neurofibromatosis type 2 diagnostic criteria to exclude LZTR1-related schwannomatosis. Neurology.

[23] Teranishi Y, Miyawaki S, Hongo H, Dofuku S, Okano A, Takayanagi S (2021). Targeted deep sequencing of DNA from multiple tissue types improves the diagnostic rate and reveals a highly diverse phenotype of mosaic neurofibromatosis type 2. J Med Genet.

[24] Subramanian A, Tamayo P, Mootha VK, Mukherjee S, Ebert BL, Gillette MA (2005). Gene set enrichment analysis: a knowledge-based approach for interpreting genome-wide expression profiles. Proc Natl Acad Sci USA.

[25] Hanzelmann S, Castelo R, Guinney J (2013). GSVA: gene set variation analysis for microarray and RNA-seq data. BMC Bioinform.

[26] Newman AM, Liu CL, Green MR, Gentles AJ, Feng W, Xu Y (2015). Robust enumeration of cell subsets from tissue expression profiles. Nat Methods.

[27] Aran D, Zicheng Hu, Butte AJ (2017). xCell/digitally portraying the tissue cellular heterogeneity landscape. Genome Biol.

[28] Yoshihara K, Shahmoradgoli M, Martínez E, Vegesna R, Kim H, Garcia WT (2013). Inferring tumour purity and stromal and immune cell admixture from expression data. Nat commun.

[29] Rapp C, Dettling S, Liu F, Ull AT, Warta R, Jungk C (2019). Cytotoxic T cells and their activation status are independent prognostic markers in meningiomas. Clin Cancer Res.

[30] Lee EJ, Kim JH, Park ES, Kim YH, Lee JK, Hong SH (2017). A novel weighted scoring system for estimating the risk of rapid growth in untreated intracranial meningiomas. J Neurosurg.

[31] Yeung J, Yaghoobi V, Miyagishima D, Vesely MD, Zhang T, Badri T (2021). Targeting the CSF1/CSF1R axis is a potential treatment strategy for malignant meningiomas. Neuro Oncol.

[32] Blume C, Dogan D, Schweizer L, Peyre M, Doll S, Picard D (2021). Integrated phospho-proteogenomic and single-cell transcriptomic analysis of meningiomas establishes robust subtyping and reveals subtype-specific immune invasion. BioRxiv.

[33] Choudhury A, Magill ST, Eaton CD, Prager BC, Chen WC, Cady MA (2022). Meningioma DNA methylation groups identify biological drivers and therapeutic vulnerabilities. Nat Genet.

[34] Wang AZ, Bowman-Kirigin JA, Desai R, Kang LI, Patel PR, Patel B (2022). Single-cell profiling of human dura and meningioma reveals cellular meningeal landscape and insights into meningioma immune response. Genome Med.

[35] Williams EA, Santagata S, Wakimoto H, Shankar GM, Barker FG, Sharaf R (2020). Distinct genomic subclasses of high-grade/progressive meningiomas: NF2-associated, NF2-exclusive, and NF2-agnostic. Acta Neuropathol Commun.

