# Supplementary material for: Meningiomas in patients with neurofibromatosis type 2 predominantly comprise ‘immunogenic subtype’ tumours characterised by macrophage infiltration
Source: Acta Neuropathol Commun. 2023 Sep 26;11:156. doi: 10.1186/s40478-023-01645-3 (PMC10521403; doi:10.1186/s40478-023-01645-3)
Supplement: Supplementary file 1 — Additional file 1. Figure S1. Flow Chart in this study; Figure S2.A: The MA plot based on the RNA sequencing in NF2 patients and sporadic NF2-altered meningiomas. B: The volcano plot based on the RNA sequencing in NF2 patients and sporadic NF2-altered meningiomas. C: The principal component analysis based on the RNA sequencing in NF2 patients and sporadic NF2-altered meningiomas. D: Gene set variation analysis (GSVA) based on c7 immunologic signature gene sets clearly distinguished 2 clusters; Figure S3. The MA plot based on the RNA sequencing in germline NF2 patients and mosaic NF2 patients; Figure S4. A: Each immunologic gene expression in NF2 patients and sporadic NF2-altered meningiomas.B: Each immunologic gene expression in ‘1p loss (-)’ and ‘1p loss (+)’. C: Each immunologic gene expression in ‘recurrence (-)’ and ‘recurrence (+)’; Figure S5. Deconvoluted score using CIBERSORT, xCell, and ESTIMATE. A: Each deconvoluted score in NF2 patients and sporadic NF2-altered meningiomas. B: Each deconvoluted score in ‘1p loss (-)’ and ‘1p loss (+)’. C: Each deconvoluted score in ‘recurrence (-)’ and ‘recurrence (+)’; Figure S6. Infiltrated cells based on deconvoluted data. A: Each infiltrated cell in NF2 patients and sporadic NF2-altered meningiomas. B: Each infiltrated cell in ‘1p loss (-)’ and ‘1p loss (+)’. C: Each infiltrated cell in ‘recurrence (-)’ and ‘recurrence (+)’; Figure S7. GSEA using single-cell data (C8): NF2 vs sporadic; Figure S8. GSEA using single-cell data (C8): non-recurrence vs recurrence; Figure S9. Quantification of immune cell infiltration by IHC. A: Quantification of immune cells in “1p loss (-)” and ”1p loss (+)”. B: Quantification of immune cells in ‘recurrence (-)’ and ‘recurrence (+)’; Figure S10. Quantification of immune cell infiltration by IHC in each case; Figure S11. A-C: The correlation analysis of each RNA-seq–derived immune metrics (CIBERSORT vs ESTIMLATE [A], xCell vs CIBERSORT [B], and ESTIMATE vs xCell [C]). D,E: The correlation a [file 40478_2023_1645_MOESM1_ESM.pdf]

Figure S1 Flow chart in this study

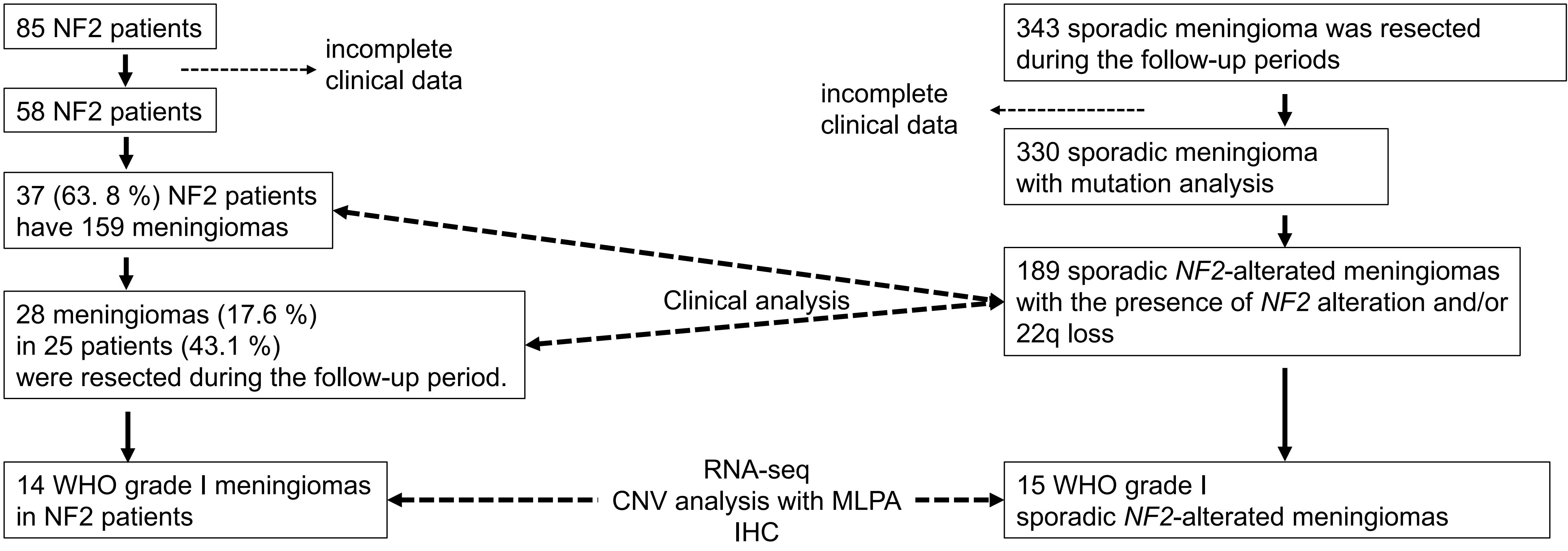

Figure S2

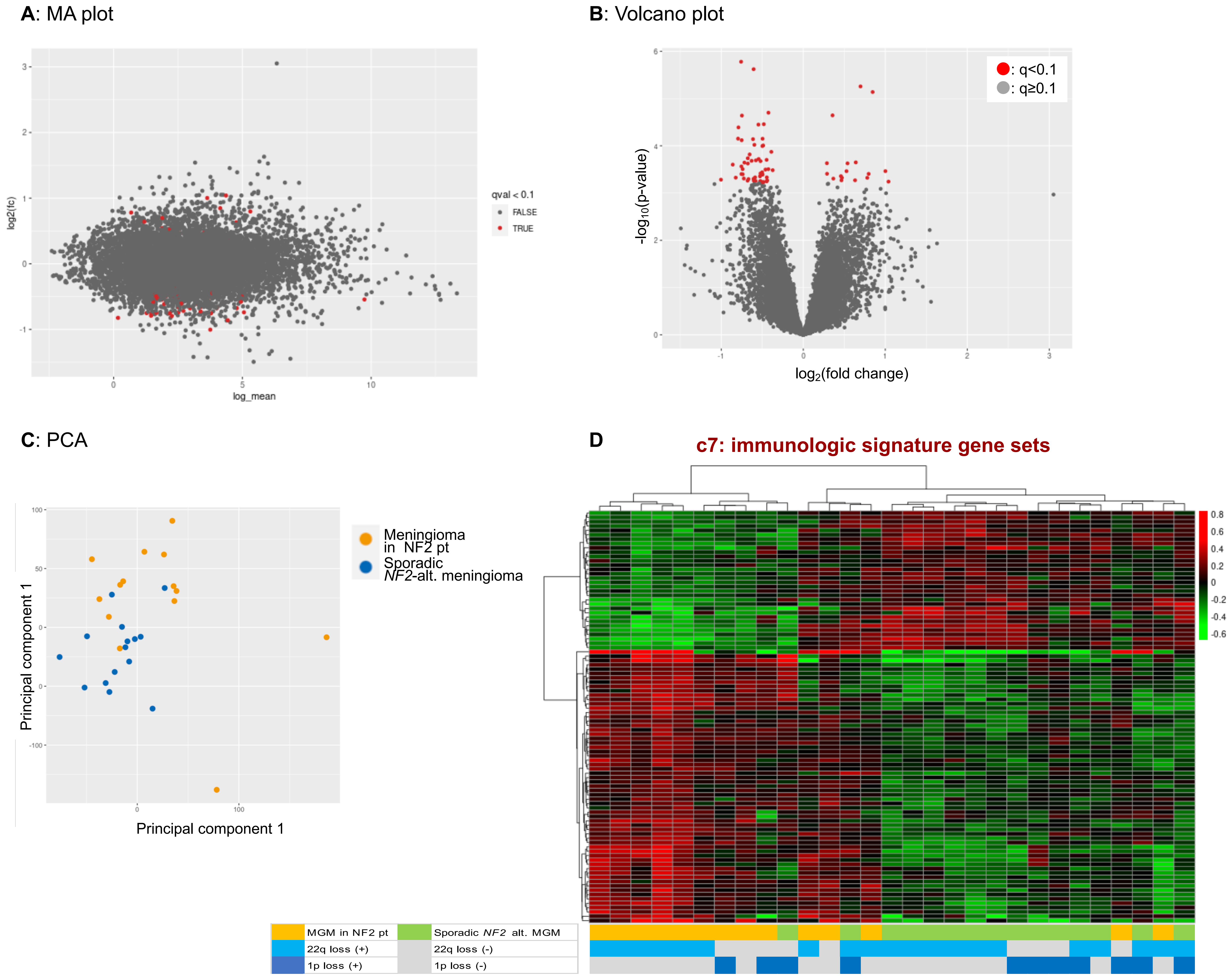
A: MA plot
log2(fc)
log_mean
qval < 0.1
FALSE
TRUE
B: Volcano plot
: q<0.1
: q≥0.1
-log10(p-value)
log2(fold change)
C: PCA
Meningioma in NF2 pt
Sporadic NF2-alt. meningioma
Principal component 1
Principal component 1
D
c7: immunologic signature gene sets
0.8
0.6
0.4
0.2
0
-0.2
-0.4
-0.6
MGM in NF2 pt
Sporadic NF2 alt. MGM
22q loss (+)
22q loss (-)
1p loss (+)
1p loss (-)

Figure S3

MA plot: germline NF2 patients (5 tumors) vs mosaic NF2 patients (9 tumors)

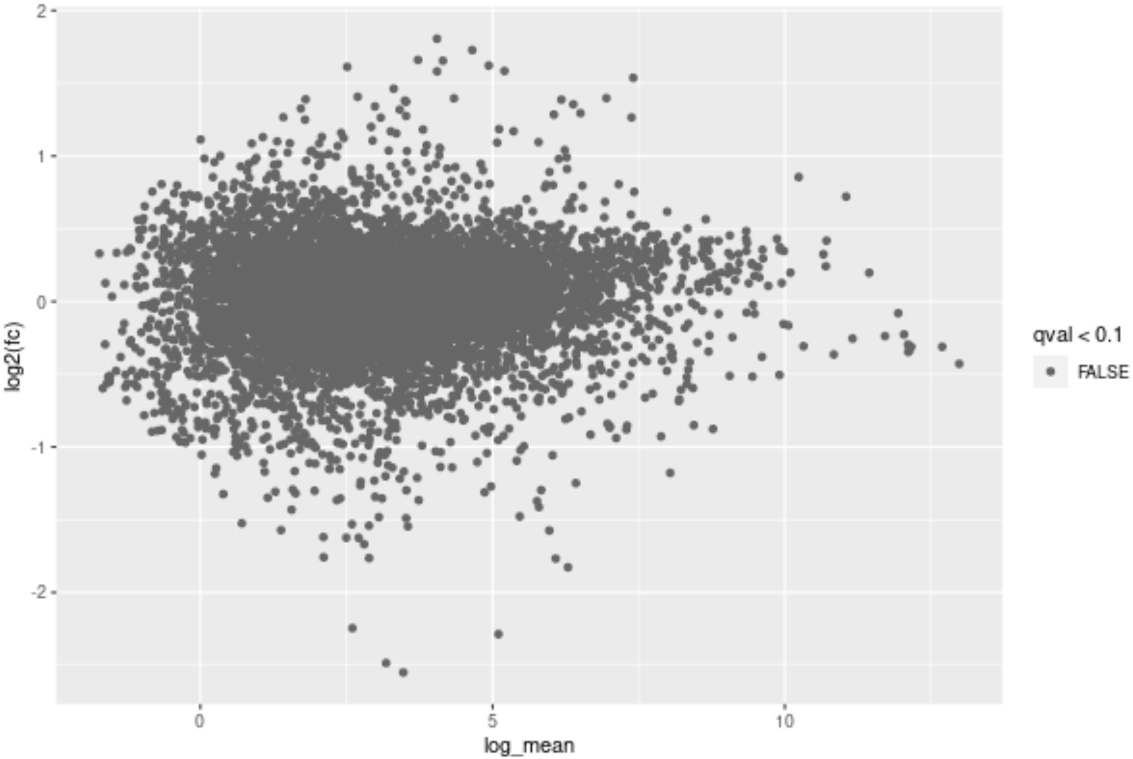

Figure S4: Each gene expression regarding immune cell

A: NF2 patient vs Sporadic NF2-alt. meningiomas

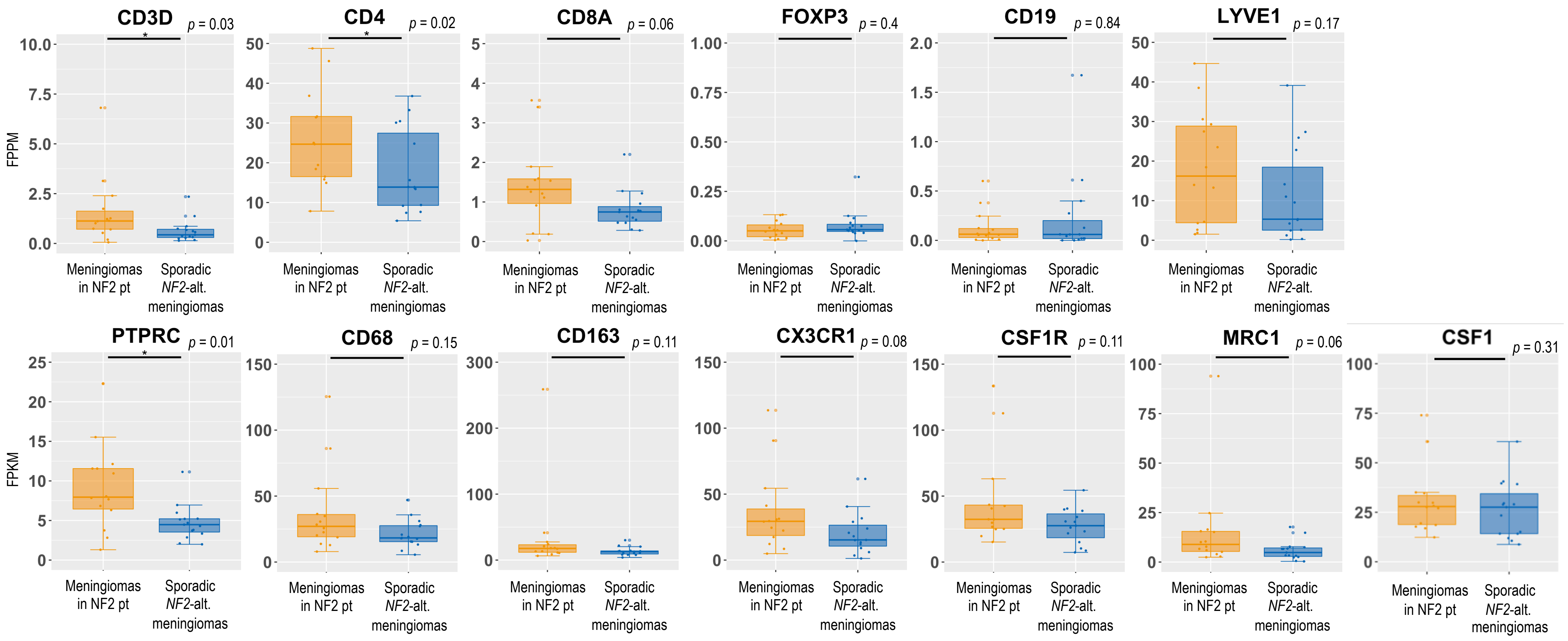

B: 1p loss(-) vs 1p loss(+)

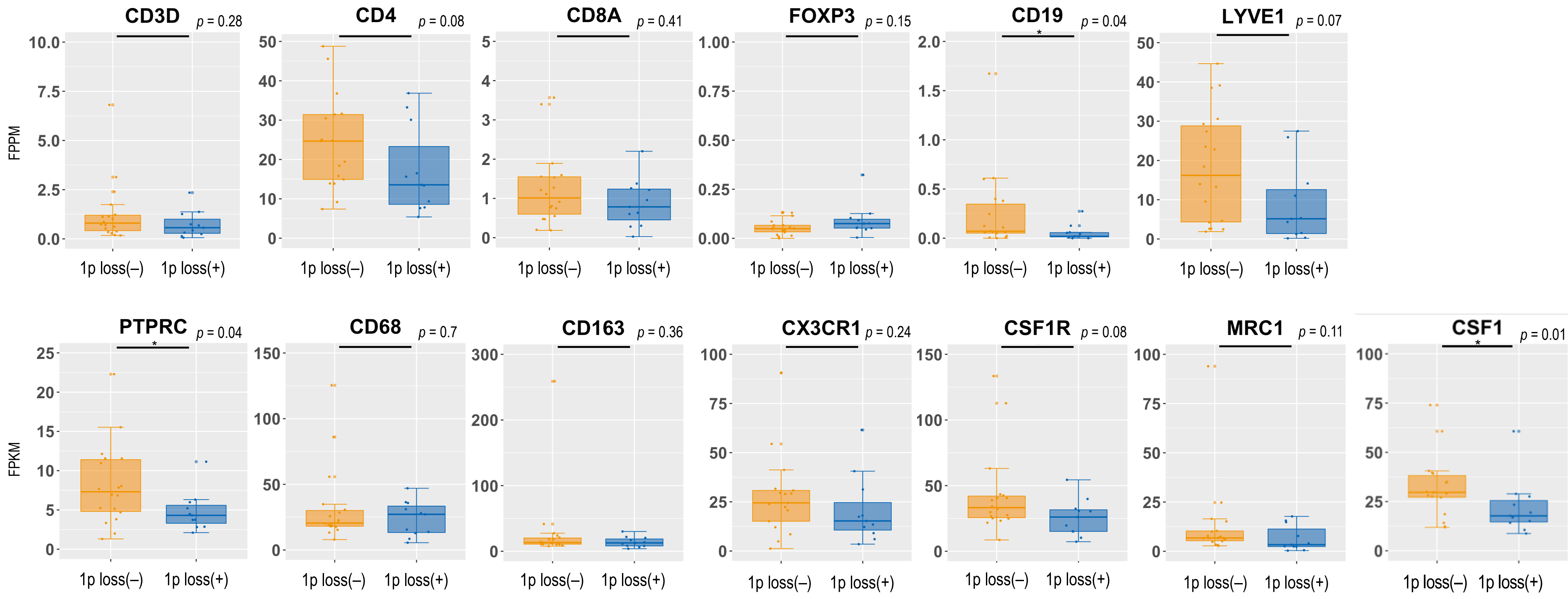

C: Recurrence (-) vs recurrence (+)

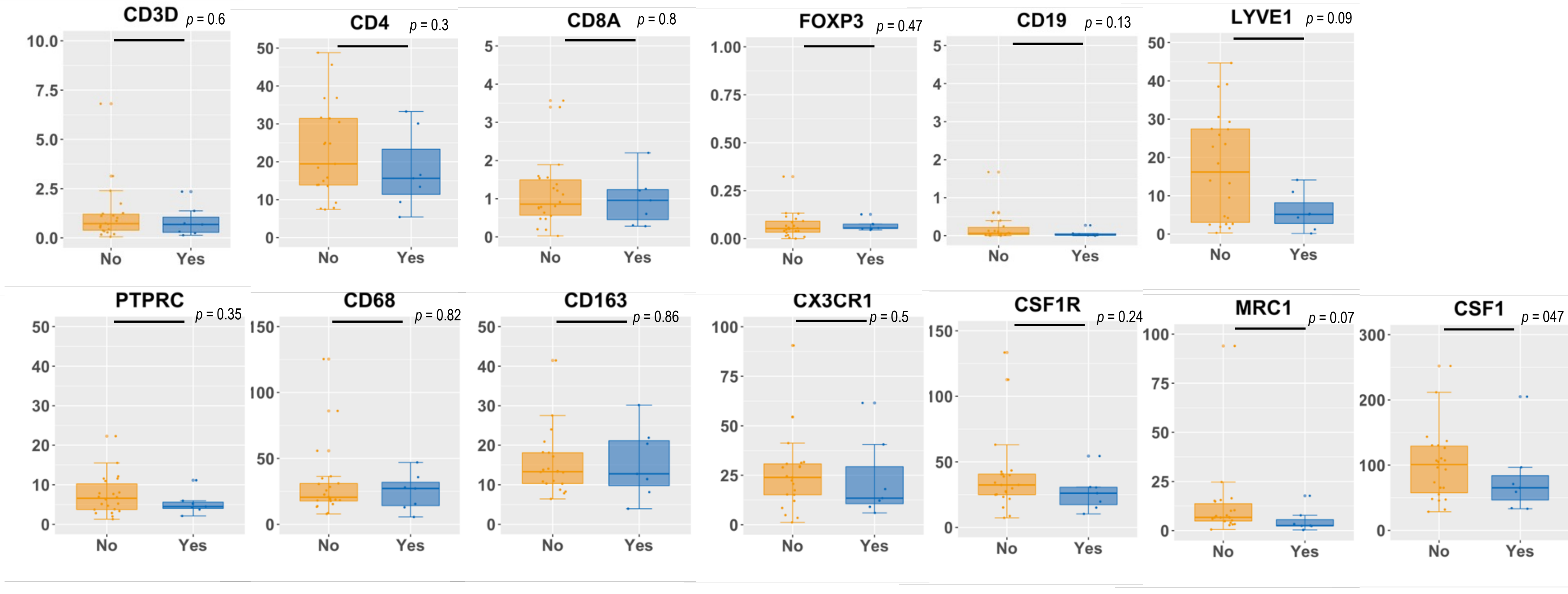

Figure S5: Each deconvulsive score

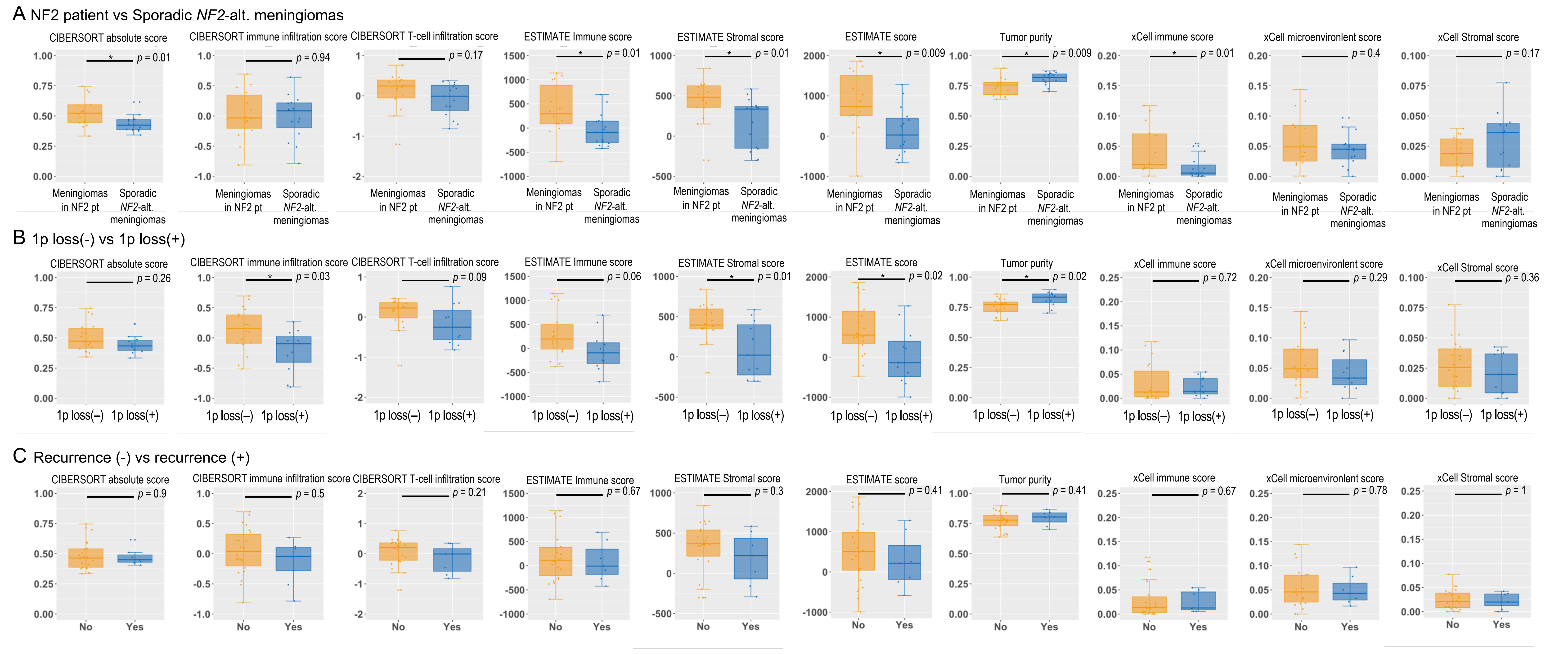

Figure S6: Each infiltration cells based on deconvulsive data

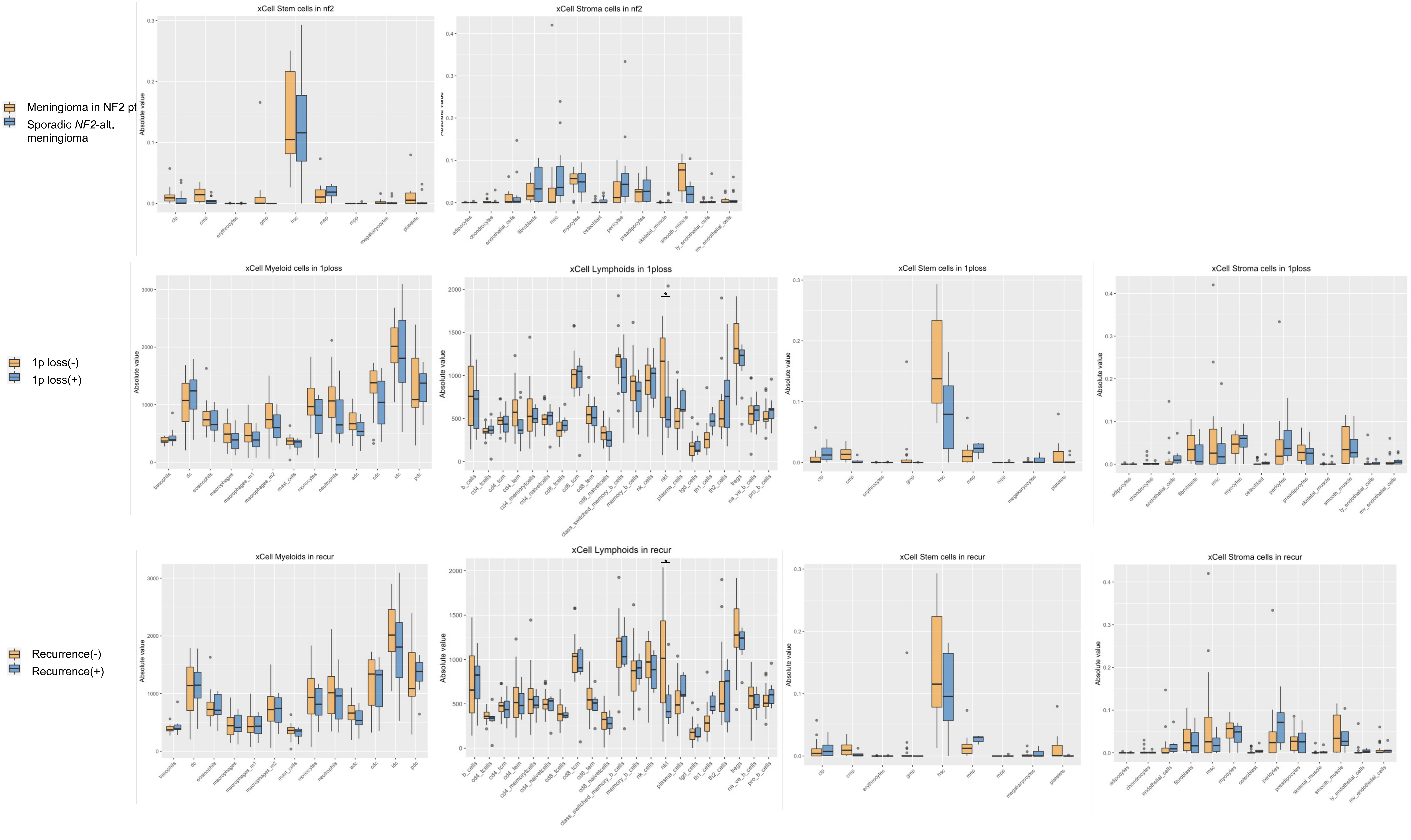

Figure S7: GSEA using single-cell data (C8): NF2 vs sporadic

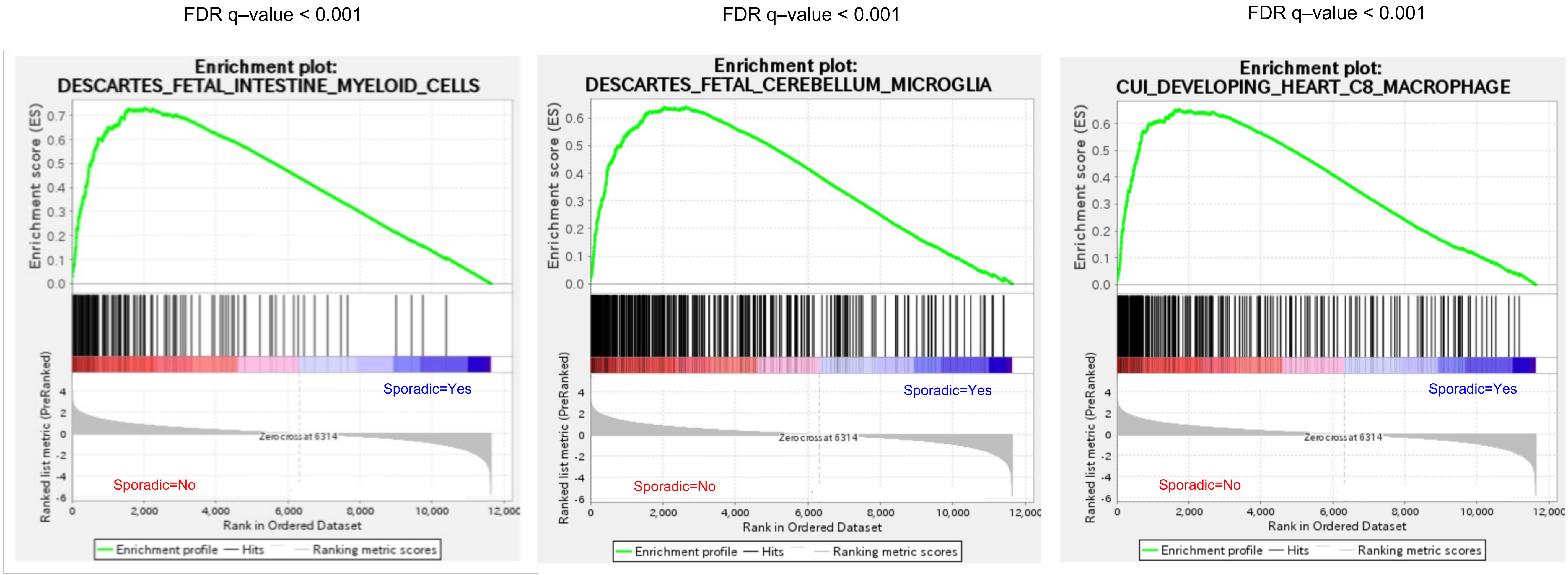

Figure S8: GSEA using single-cell data (C8): non-recurrence vs recurrence

FDR q–value = 0.026

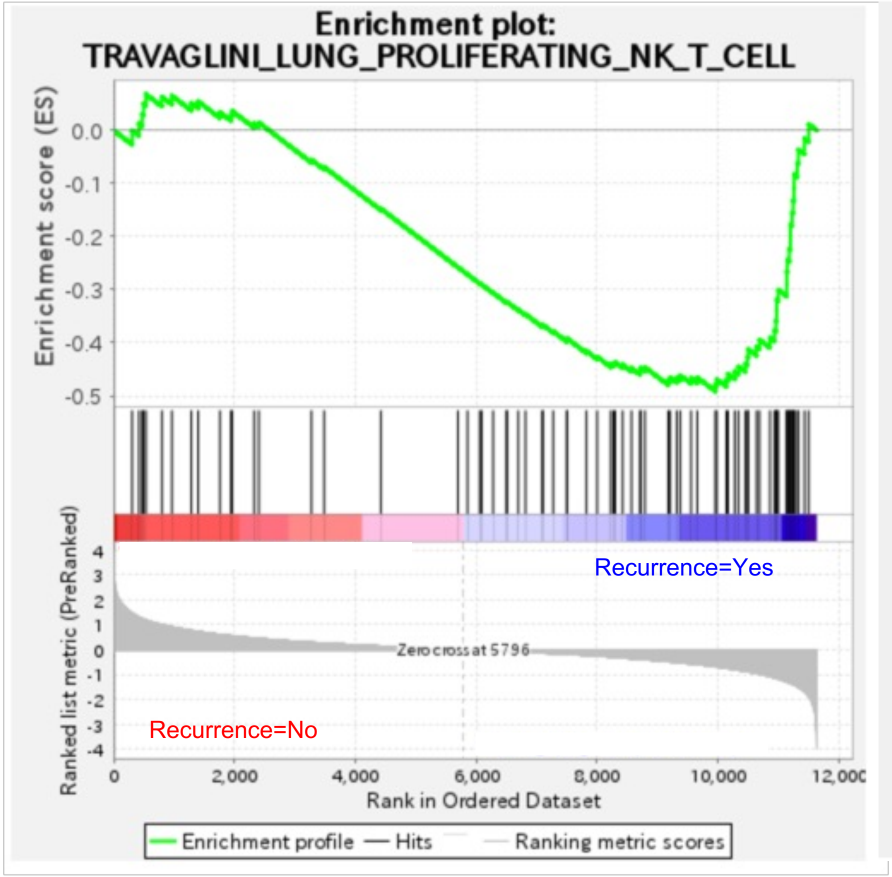

FDR q–value = 0.034

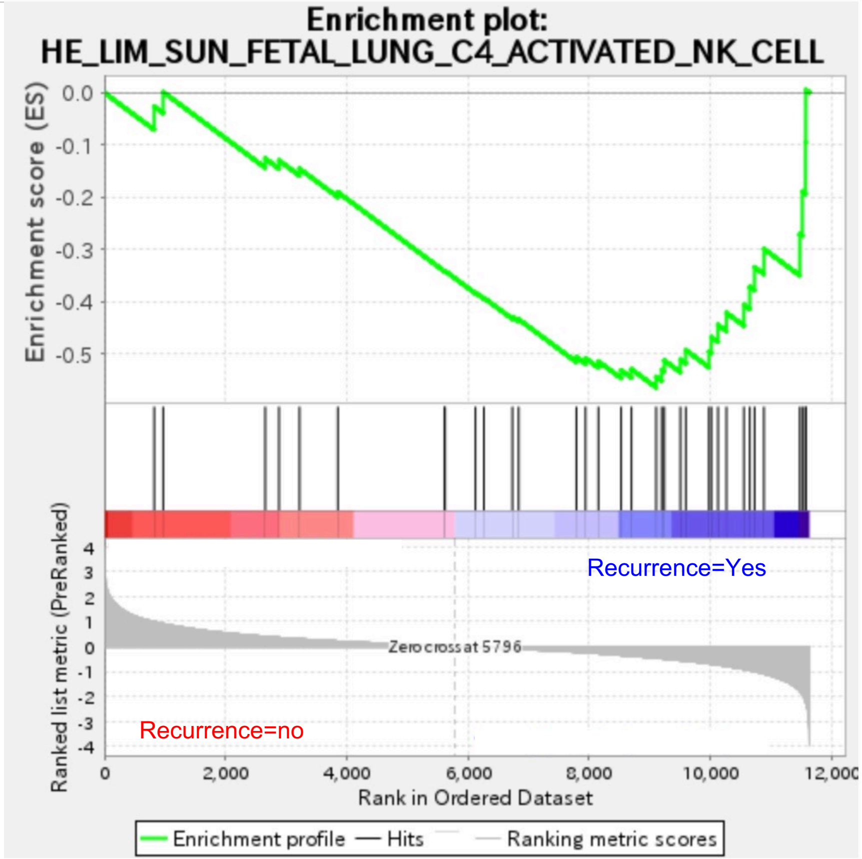

Figure S9: Quantification of immune cell infiltration by IHC depending in CNV and recurrence

A: 1p loss(-) vs 1p loss(+)

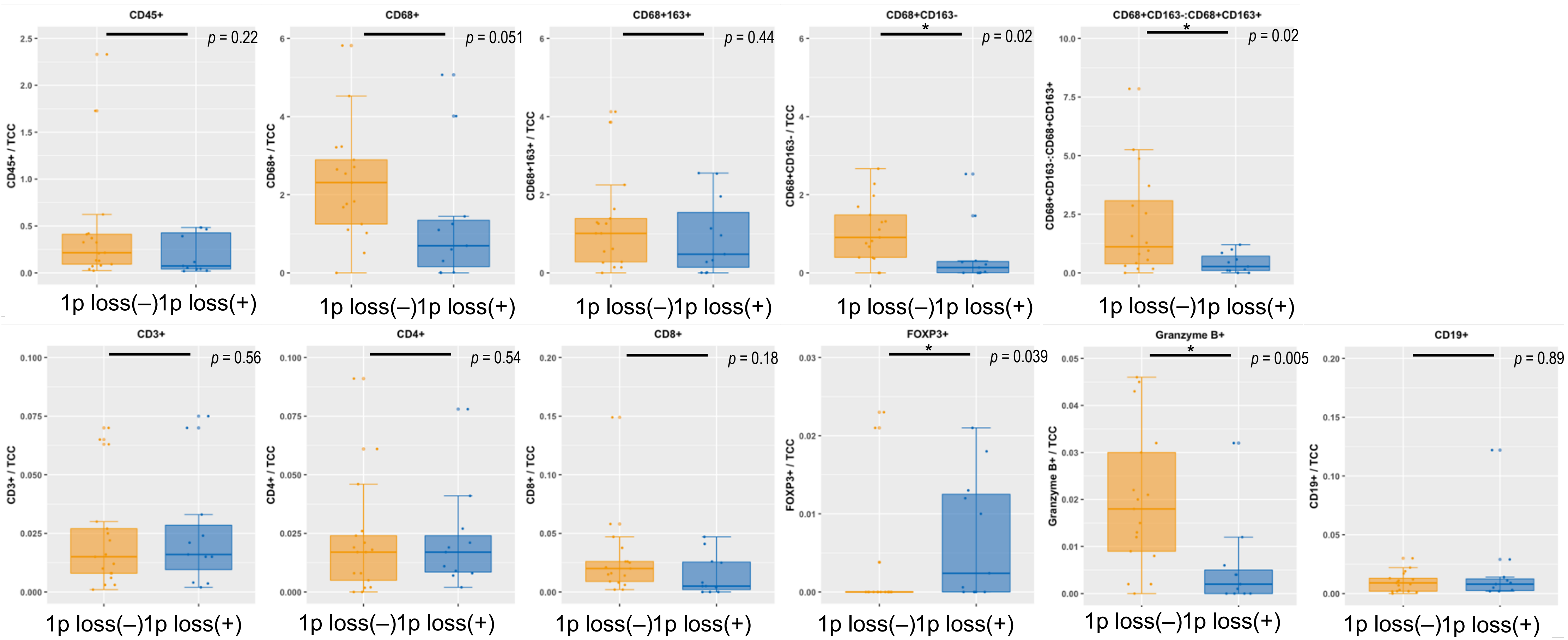

B Recurrence (-) vs recurrence (+)

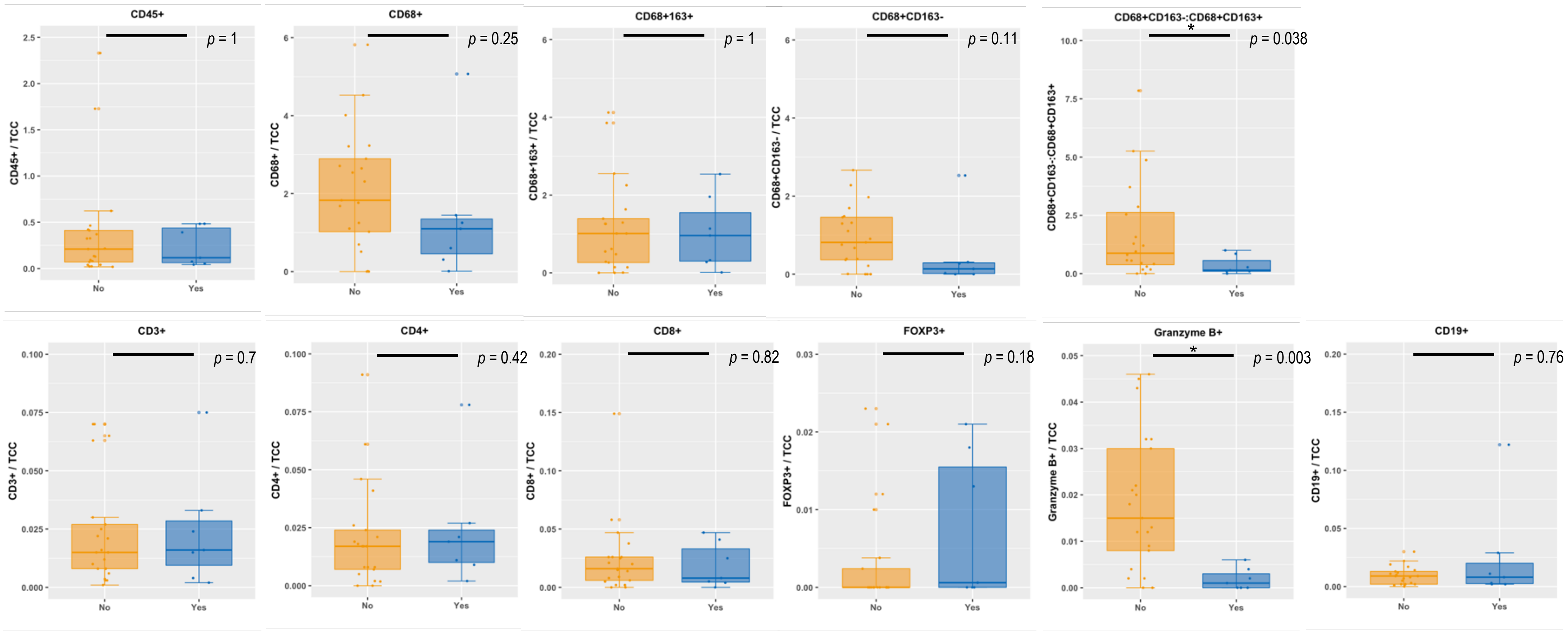

Figure S10: IHC results depending on each case

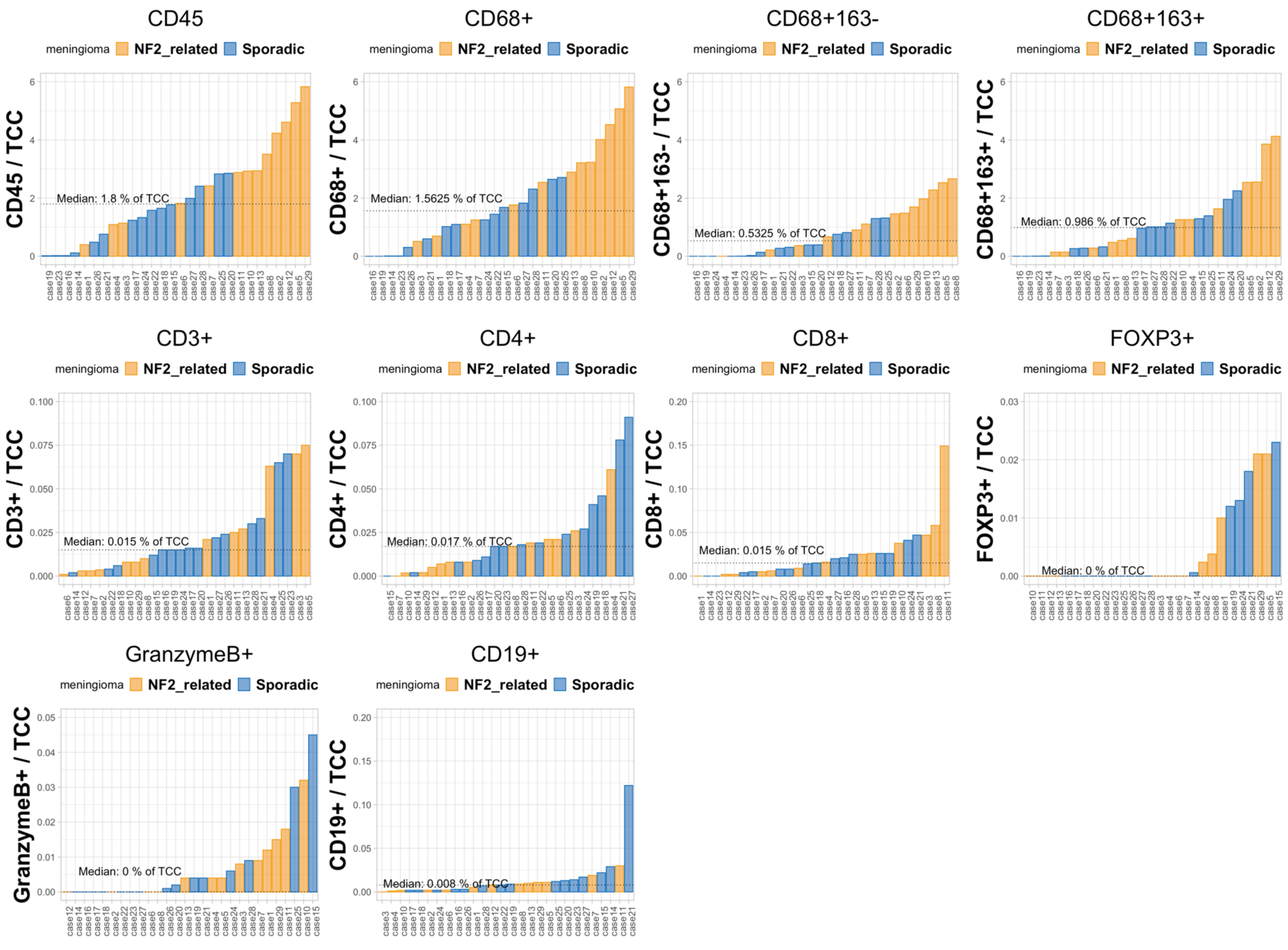

Figure S11

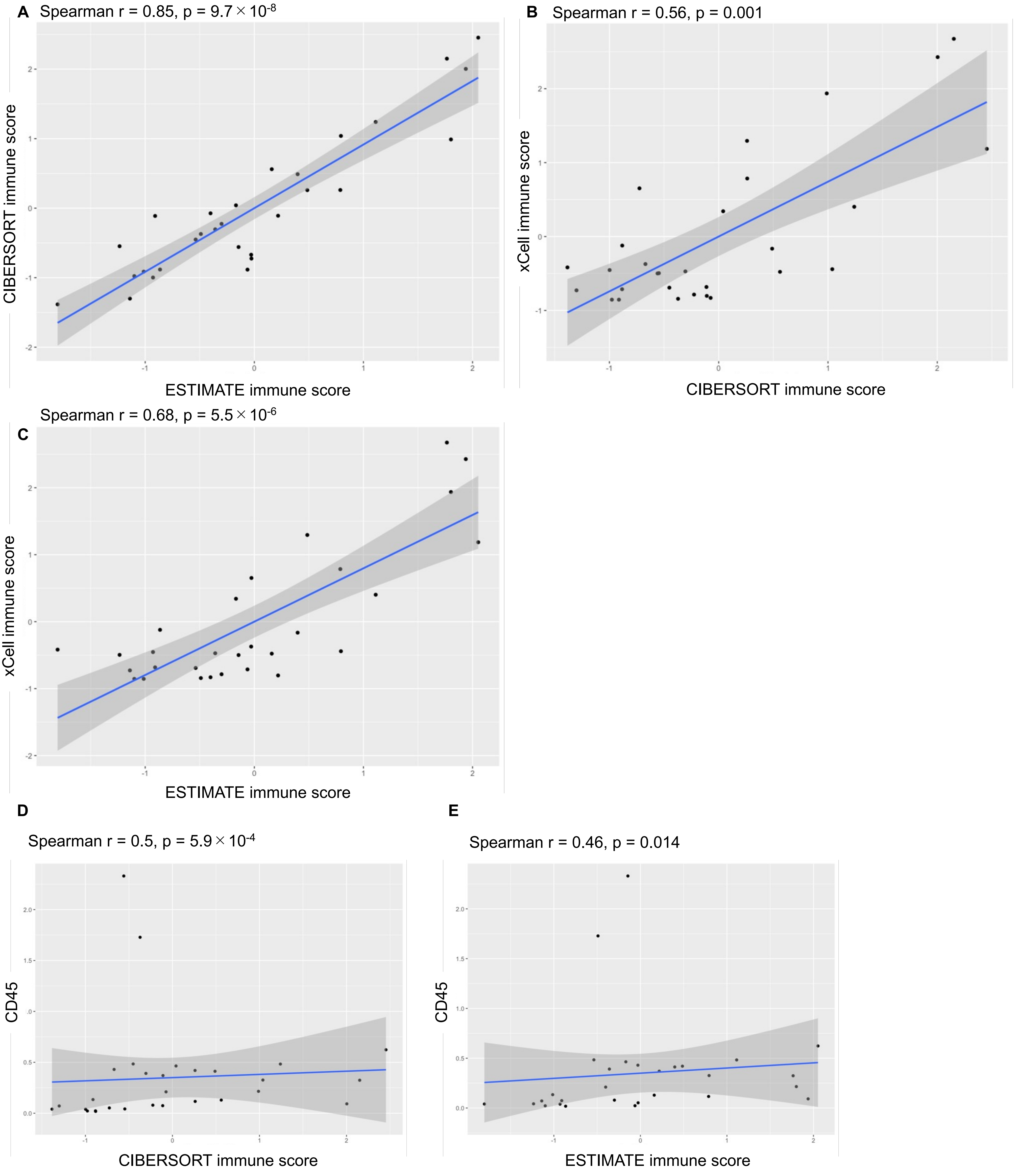
A
Spearman r = 0.85, p = 9.7 × 10-8
CIBERSORT immune score
ESTIMATE immune score
B
Spearman r = 0.56, p = 0.001
xCell immune score
CIBERSORT immune score
C
Spearman r = 0.68, p = 5.5 × 10-6
xCell immune score
ESTIMATE immune score
D
Spearman r = 0.5, p = 5.9 × 10-4
CD45
CIBERSORT immune score
E
Spearman r = 0.46, p = 0.014
CD45
ESTIMATE immune score

Figure S12: Gene expression regarding “immunogenic subtype” of meningioma

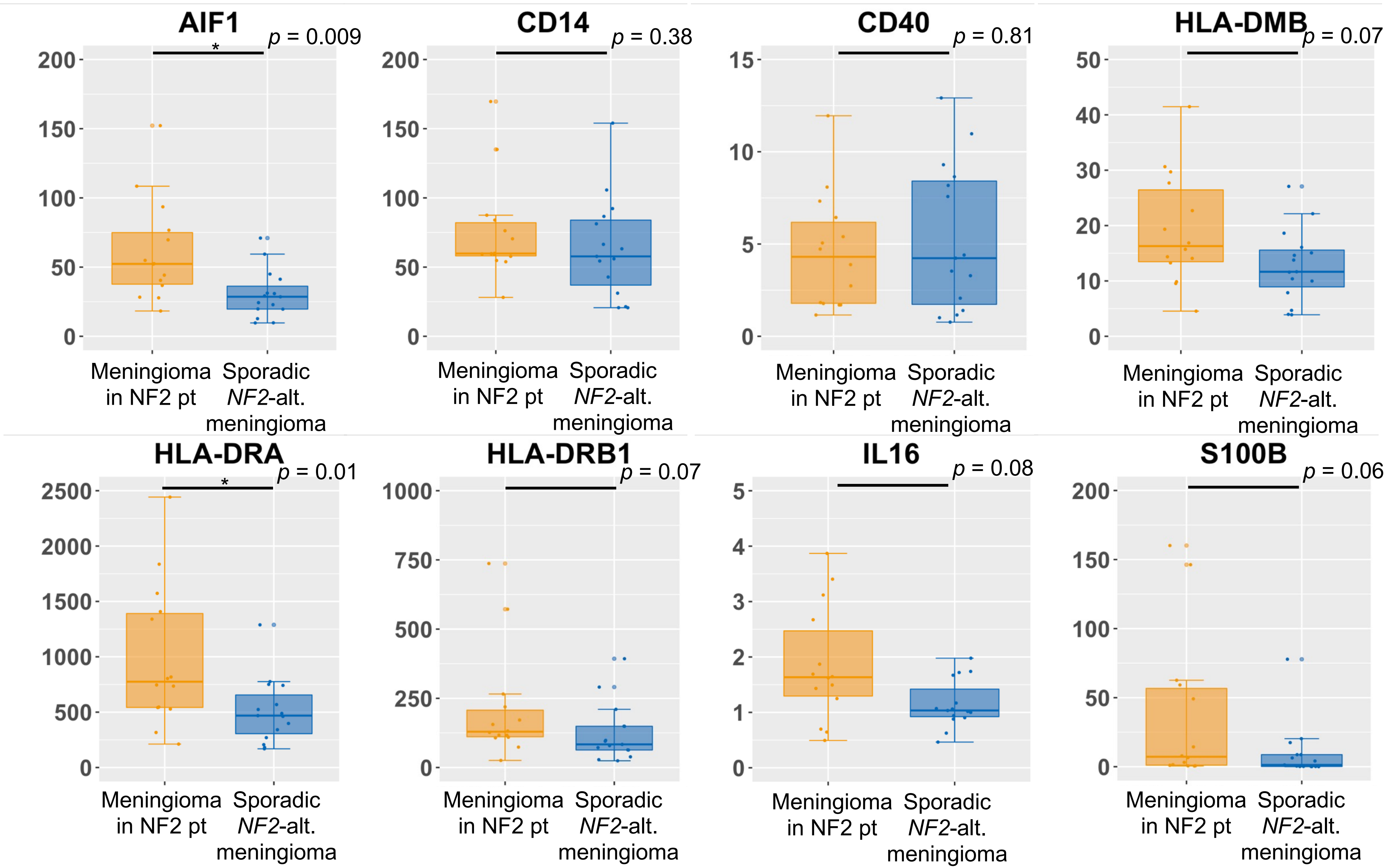

Figure S1.
Flow Chart in this study

Figure S2.
A: The MA plot based on the RNA sequencing in NF2 patients and sporadic *NF2*-altered meningiomas.
B: The volcano plot based on the RNA sequencing in NF2 patients and sporadic *NF2*-altered meningiomas.
C: The principal component analysis based on the RNA sequencing in NF2 patients and sporadic *NF2*-altered meningiomas.
D: Gene set variation analysis (GSVA) based on c7 immunologic signature gene sets clearly distinguished 2 clusters.

Figure S3.
The MA plot based on the RNA sequencing in germline NF2 patients and mosaic NF2 patients.

Figure S4.
A: Each immunologic gene expression in NF2 patients and sporadic *NF2*-altered meningiomas.
B: Each immunologic gene expression in '1p loss (-)' and '1p loss (+)'.
C: Each immunologic gene expression in 'recurrence (-)' and 'recurrence (+)'.

Figure S5. Deconvoluted score using CIBERSORT, xCell, and ESTIMATE.
A: Each deconvoluted score in NF2 patients and sporadic *NF2*-altered meningiomas.
B: Each deconvoluted score in '1p loss (-)' and '1p loss (+)'.
C: Each deconvoluted score in 'recurrence (-)' and 'recurrence (+)'.

Figure S6. Infiltrated cells based on deconvoluted data.
A: Each infiltrated cell in NF2 patients and sporadic *NF2*-altered meningiomas.
B: Each infiltrated cell in '1p loss (-)' and '1p loss (+)'.
C: Each infiltrated cell in 'recurrence (-)' and 'recurrence (+)'.

Figure S7. GSEA using single-cell data (C8): NF2 vs sporadic.

Figure S8. GSEA using single-cell data (C8): non-recurrence vs recurrence.

Figure S9. Quantification of immune cell infiltration by IHC.
A: Quantification of immune cells in "1p loss (-)" and "1p loss (+)".
B: Quantification of immune cells in 'recurrence (-)' and 'recurrence (+)'.

Figure S10. Quantification of immune cell infiltration by IHC in each case.

Figure S11.
A-C: The correlation analysis of each RNA-seq–derived immune metrics (CIBERSORT vs ESTIMLATE [A], xCell vs CIBERSORT [B], and ESTIMATE vs xCell [C]).
D,E: The correlation analysis of IHC-derived and RNA-seq–derived measures of leukocyte infiltration (CD45 cells vs CIBERSORT absolute score [D], and ESTIMATE immune score [E]).

Figure S12.
Gene expression regarding ‘immunogenic subtype’ of meningiomas
